# A molecular rheostat of ABA signaling coordinates the trade-off between stem growth and drought adaptation

**DOI:** 10.1186/s13059-026-04228-2

**Published:** 2026-08-04

**Authors:** Chenfei Lv, Yuling He, Donghai Zhang, Mengjiao Zhang, Mingjia Yuan, Leishi Zhong, Chongde Tian, Jiaxuan Zhou, Rui Huang, Zhuoying Jin, Peng Li, Wenjie Lu, Liang Xiao, Jinlong Li, Mingyang Quan, Zhonghai Li, Rui Zhao, Liang Du, Li-Jun Liu, Deqiang Zhang, Qingzhang Du

**Affiliations:** 1 State Key Laboratory of Tree Genetics and Breeding, College of Biological Sciences and Technology, Beijing Forestry University, No. 35, Qinghua East Road, Beijing, 100083 People’s Republic of China; 2 National Engineering Research Center of Tree Breeding and Ecological Restoration, College of Biological Sciences and Technology, Beijing Forestry University, No. 35, Qinghua East Road, Beijing, 100083 People’s Republic of China; 3 Key Laboratory of Genetics and Breeding in Forest Trees and Ornamental Plants, Ministry of Education, College of Biological Sciences and Technology, Beijing Forestry University, No. 35, Qinghua East Road, Beijing, 100083 People’s Republic of China; 4 College of Forestry, State Forestry and Grassland Administration Key Laboratory of Silviculture in Downstream Areas of the Yellow River, Shandong Agricultural University, Tai’an 271018, Shandong, People’s Republic of China

**Keywords:** ABA-CTK signal antagonism, HD-ZIP, Hormonal rheostat, Transgenerational inheritance, CRISPR-Cas9, *Populus*

## Abstract

**Background:**

Plants balance vegetative growth and stress responses through antagonistic phytohormone crosstalk, notably between abscisic acid (ABA) and cytokinin. However, the genetic architecture and molecular mechanisms driving this adaptive trade-off, particularly in perennial trees, remain elusive.

**Results:**

Integrating genome-environment and genome-wide association studies across 302 *Populus tomentosa* accessions, we identify *PtoAG1* as a pleiotropic regulator of climate adaptation and stem growth. A naturally occurring frameshift mutation abolishes its repressor function, driving geographical niche differentiation by enhancing growth in humid habitats. Mechanistically, *PtoAG1* acts as a molecular rheostat. Under drought, ABA-activated PtoSnRK2.2 phosphorylates PtoMYB84 to transactivate *PtoAG1*. PtoAG1 then represses the cytokinin-signaling regulator *PtoRR12*, consequently releasing its inhibition of PtoSnRK2.2. This positive feedback further suppresses vegetative growth under stress while amplifying ABA responses via PtoAREB3. Concurrently, PtoAG1 directly represses *PtoAREB3* as a transcriptional brake to maintain signaling homeostasis. Evolutionary analyses reveal that *PtoMYB84* and *PtoAG1* constitute a tightly linked *cis*-regulatory module conserved across eudicots. Functional analysis on *AG1* orthologs in poplar, *Arabidopsis*, rice, and tomato demonstrates conserved cross-species utility: loss-of-function increases vegetative biomass but overexpression significantly boosts drought resilience.

**Conclusions:**

Our study identifies a hierarchical crosstalk mechanism bridging ABA and cytokinin signaling to optimize environmental fitness. The evolutionarily conserved *MYB84*-*AG1 cis*-regulatory module establishes a versatile, translatable genetic target for precision breeding of climate-resilient and high-yielding crops.

**Supplementary Information:**

The online version contains supplementary material available at https://doi.org/10.1186/s13059-026-04228-2.

## Background

Over millennia of climatic upheaval, plants have evolved sophisticated molecular switches to dynamically balance growth-stress tolerance transitions. Drought stress constitutes a primary constraint on global terrestrial ecosystem productivity, making enhanced drought resilience in trees critical for forest adaptability and sustainable plantation biomass production [1, 2]. Consequently, deciphering the signal transduction mechanisms that orchestrate rapid growth-to-drought tolerance transitions will facilitate the rational design of climate-resilient cultivars, thereby enhancing carbon sequestration efficiency and yield stability maintenance.

As master integrators of environmental cues and endogenous developmental programs, phytohormones orchestrate growth phase transitions and reconfigure metabolic networks in plants [3]. The ABA signal perception module (PYRABACTIN RESISTANCE PROTEINS (PYR)/PYR-LIKE PROTEINS (PYL)/REGULATORY COMPONENTS OF ABA RECEPTORS (RCAR)) recruits clade A protein phosphatase type 2Cs (PP2Cs) to relieve autoinhibition of SNF1-RELATED KINASE 2 (SnRK2) kinase. Subsequent SnRK2-mediated phosphorylation cascades reprogram physiological responses through substrate network activation [4–6]. Subthreshold ABA concentrations launch a self-reinforcing circuit: primary activation of PYR/PYL-RCAR receptors triggers SnRK2-dependent transcriptional induction of NCED3 and ABA biosynthetic enzymes, catalyzing exponential accumulation of endogenous ABA that exceeds initial stimulus intensity by orders of magnitude [7, 8]. Notably, PYL receptors stabilize ABI5 transcription factor to potentiate ABA-responsive gene expression, while ABI5 reciprocally upregulates PYL expression, thereby establishing a bistable positive feedback loop within the ABA signaling cascade [9]. This co-amplification module drives accelerated stress response kinetics through SnRK2 kinase phosphorylation cascades, representing a phylogenetically constrained adaptation to environmental stochasticity that optimizes fitness under transient resource constraints.

However, prolonged ABA signaling intensification disrupts metabolic homeostasis and severely suppresses plant growth [10]. Chronically elevated endogenous ABA level induces receptor desensitization, impairing developmental programs during stresses [11]. Such autoregulatory constrains evolved in early terrestrial plants to optimize desiccation tolerance while enabling rapid terrestrial colonization [12]. Crucially, transcriptional suppression of PYL receptors prevents hyperaccumulation under stress conditions [7, 13], meanwhile key ABA-responsive genes show transient activation followed by timed repression [14–16]. These layered controls constitute an evolutionarily fine-tuned braking system maintaining ABA signaling within physiological thresholds to dynamically reconcile environment adaptation with growth homeostasis.

ABA and CTK exhibit antagonistic crosstalk in regulating plant development and abiotic stress responses [17]. CTK signaling operates through a two-component phosphorelay system (TCS) comprising histidine kinases (HKs), histidine phosphotransfer proteins (HPs), and response regulators (RRs), which is suppressed by ABA under osmotic stress [18, 19]. This inhibition is further evidenced by the restoration of ABA-blocked root growth in *ARR5* transgenic plants via *snrk2.2/2.3* mutations [20, 21], while ABSCISIC ACID INSENSITIVE 4 (ABI4) represses type-A ARR to control seed germination [22]. Conversely, CTK deficiency (*ahk2,3*, *CKX6*-overexpression lines) and *arr1/10/12* knockout exhibit enhanced drought tolerance but compromises growth [19, 23, 24]. Despite these advances, the central regulators of ABA-CTK antagonism during environmental adaptation remain unidentified.

Homeodomain-leucine zipper (HD-ZIP) transcription factors are plant-specific regulators of growth-local adaptation, which represent key targets for plant improvement [25, 26]. The HD-ZIP Ⅱ subfamily uniquely possesses two signature motifs LxLxL and CPSCE, enabling functional specialization. The CPSCE motif mediates environmental signal perception [27], while the LxLxL motif interacts with TOPLESS co-repressors to confer transcriptional inhibition capacity [28]. HD-ZIP Ⅱ members (e.g., *HAT1/4*, *AtHB4*) integrate hormonal crosstalk by modulating auxin/brassinosteroid/gibberellin signaling [29, 30]. Crucially, SnRK2-I kinases phosphorylate HB1 in an ABA-dependent manner to maintain osmotic homeostasis [31], while *hat1/hat3* loss-of-function mutations reduce ABA responsiveness and confer drought tolerance [30]. These orthogonal regulatory axes establish HD-ZIP II factors as core processors in plant abiotic stress adaptation.

This study identifies a major QTL ADAPTATION-GROWTH 1 (qAG1) on Chr.07 of *Populus tomentosa,* in which the core gene *PtoAG1*, a novel HD-ZIP II gene that functions as a rheostat for ABA signaling. Within qAG1, *PtoAG1* and its proximal transcription factor PtoMYB84 form a conserved dicot module to mediate crosstalk between ABA and CTK pathways. Drought induces this module, which inhibits CTK signaling factor PtoRR12, thereby lifting an auxiliary brake to fine-tune and facilitate PtoSnRK2.2 autophosphorylation. Activated PtoSnRK2.2 phosphorylates and activates both PtoMYB84 and PtoAREB3, creating an ABA signaling positive feedback loop to rapidly boost drought tolerance. Concurrently, PtoAG1 directly represses *PtoAREB3* to attenuate ABA signaling, forming a braking mechanism that tightly controls ABA signaling homeostasis. This dual-track regulatory loop enables plants to dynamically transition between growth and drought stress states, thereby influencing xylem anatomy and biochemical responses over evolutionary timescales. A natural frameshift mutation in *PtoAG1* exhibits stable transgenerational inheritance across hybrid populations. Functional validation through CRISPR/Cas9 knockout and overexpression of *AG1* orthologs in *Arabidopsis*, rice, and tomato demonstrated cross-species functional conservation in either growth vigor or drought resistance without mutual tradeoffs.

## Results

### Integrative genomics reveals pleiotropic loci for climate adaptation and growth across natural populations of P. tomentosa

**Fig. 1:**
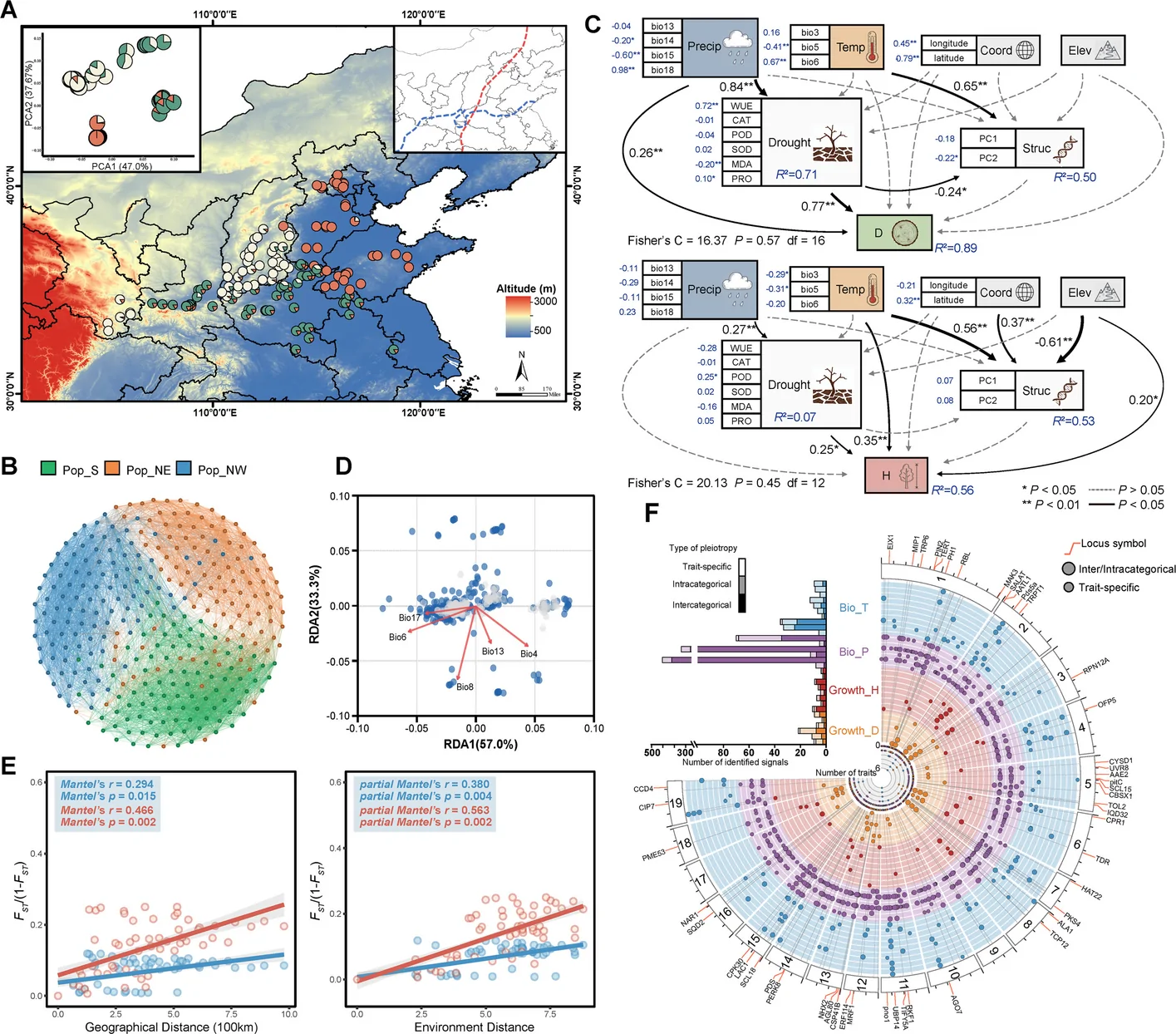
The distribution of poplar populations influences its growth and adaptability. **A** Geographic distribution of 302 *P. tomentosa* accessions (circles) where colors represent ancestral components inferred by ADMIXTURE (according to the substructure at K = 3). Left inset: Principal component analysis (PCA) plots of the first two components of the genetic structure for *P. tomentosa* accession. **B** Cluster analysis based on the correlations between 19 environmental variables and growth traits over 8 years. A force-directed layout based on the Fruchterman-Reingold model was performed on 302 *P. tomentosa* accessions. Edge weight = 0.9. **C** Piecewise structural equation modeling (SEM) of precipitation, temperature, elevation, coordinate, genetic structure of 104 poplar individuals and six physiological traits of these poplar individuals before and after drought treatment on plant basal diameter and height. **D** The Projection of loci and environmental variables along the first two RDA axes. Visualize the adaptive variants (blue, 546 loci) in the RDA. **E** Isolation-by-distance analyses (Mantel test, two-sided) and isolation-by-environment analyses (partial Mantel test, two-sided, controlling for the effect of geographic distance) based on neutral (blue dots and line) and adaptive variants (red dots and line). The shadow of linear regression represents the 95% confidence interval. Source data are provided (Additional file 1: Fig. S1 and Additional file 2: Table S2). **F** The outer circos plot represents the identified loci and their pleiotropy. Each dot indicates a trait-associated locus. Larger dots emphasize a pleiotropic association. Each radial line links all dots corresponding to an intercategorical pleiotropic locus to a locus symbol. The inner circos plot shows the count of associated traits for each intercategorical pleiotropic locus. The stacked plot represents the number of signals identified for each trait, grouped by the type of pleiotropy

Our prior work revealed significant climatic-zone divergence in *P. tomentosa* dynamic growth trajectories [32]. Population genomic analyses of 302 accessions demonstrated the spatial genetic structure congruent with biogeographical gradients (Fig. 1A), indicating genetic underpinnings of climatic adaptation. Fruchterman-Reingold clustering based on 19 local bioclimatic variables and 8-year stem dynamic growth traits yielded a topology congruent with the genetic structure (Fig. 1B; Additional file 2: Table S1). This spatial concordance demonstrates concerted environmental-genetic drivers of growth divergence. We further explored these direct and indirect effects on poplar growth using structural equation models (SEMs) incorporating environmental factors and plant physiological responses (Fig. 1C). These models explained 89% and 56% of the total variation in basal diameter and height of stem, respectively. Consistent with our previous report [32], temperature demonstrated a significant direct effect on vertical growth (standardized total coefficient = 0.35; Additional file 1: Fig. S1A). Moreover, the results highlight that physiological responses mediated by hydrological variation play a predominant role in directly regulating radial growth in poplar (standardized total coefficient = 0.77) within the context of local adaptation assessment. These structural equation modeling (SEM) outcomes align with the complex relationships identified by the Fruchterman–Reingold network models and support the hypothesis that both plant growth and local niche differentiation are determined by the environmental modulation of plant physiological responses.

We integrated genome-environment association (GEA) and longitudinal growth genome-wide association study (GWAS) to dissect the pleiotropic genetic architecture of adaptive growth. Two complementary GEA approaches were employed to identify the environment-related genetic variation, including a latent factor mixing model (LFMM) by 19 bioclimatic variables and a Redundancy analysis (RDA) with permutation-based significance testing [33]. Following variable selection by gradient forest analysis (Spearman |r|< 0.8 threshold for multicollinearity control), five non-redundant environmental predictors were retained (Additional file 1: Fig. S1B, C). The first two RDA axes explained 90.4% of constrained variance (Fig. 1D; Additional file 1: Fig. S1D). Integration of LFMM and RDA outliers (|SD|> 3) identified 546 adaptive variants (Additional file 2: Table S2), exhibiting significantly stronger isolation-by-distance (IBD) and isolation-by-environment (IBE) patterns than neutral loci (Mantel test and partial Mantel test; Fig. 1E). Concurrently, GWAS of longitudinal height and diameter-at-breast height (16 traits over 8-year growth; EMMAX model) detected 110 significant association loci. Colocalization analysis revealed seven pleiotropic loci that jointly govern climate adaptation and growth trajectories (Fig. 1F; Additional file 2: Table S3). These results demonstrate how climatic gradients spatially structure adaptive divergence and growth optima in *P. tomentosa*.

### Fine-mapping identifies PtoAG1 frameshift allele conferring growth gain and biogeographical divergence

**Fig. 2:**
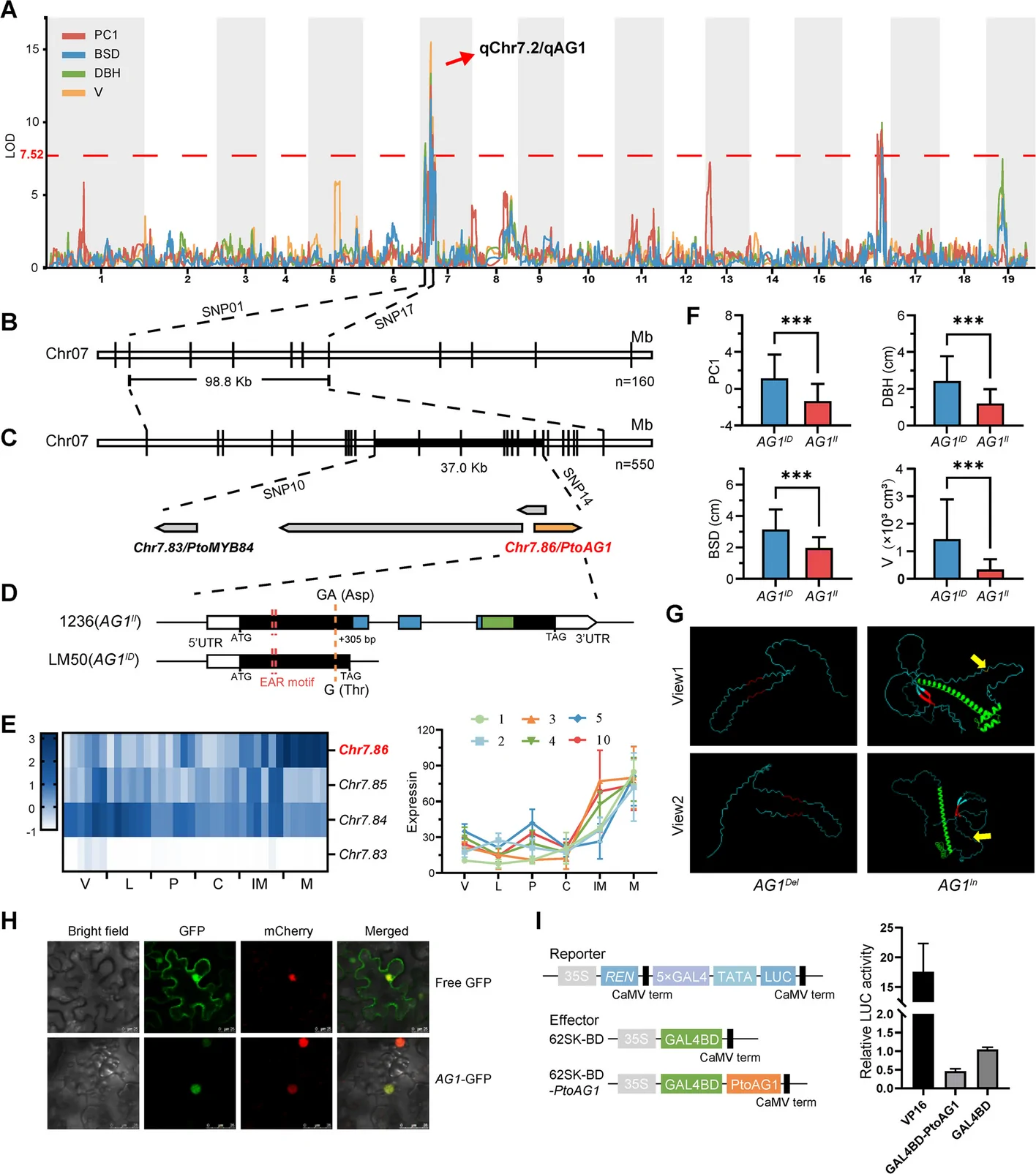
Fine mapping of the growth QTL in *P. tomentosa* and the identification of *PtoAG1*. **A** Scanning of the growth QTL qChr7.2 (qAG1) that are significantly associated with four growth traits in a pseudo-testcross population. **B** QTL analysis narrowed qAG1 locus to a 98.8 Kb region between SNP01 and SNP17 on chromosome 7. **C** Positional cloning narrowed qAG1 locus to a 37.0 kb region between SNP10 and SNP14. The orange fragment indicates the candidate gene. **D** Structure of the *PtoAG1* alleles from “1236”and “LM50” of *P. tomentosa.* Blue rectangle, homeodomain; Green rectangle, leucine zipper domain; Black rectangle, the other exons. The orange dotted line represents the position of the natural mutation *AG1*^*In/Del*^. **E** The heatmap represents the expression patterns of candidate genes in different tissues and was based on 3 biological replicates. The line chart depicts the expression patterns of *PtoAG1* in six tissues across six growth years. V, bud; L, leaf; P, phloem; C, cambium; IM, immature xylem; M, xylem. **F** Principal component 1 (PC1) of 11 growth traits, diameter at breast height (DBH), basal stem diameter (BSD) and volume of timber (V) of *AG1*^*In/Del*^ in the pseudo-testcross population (error bars, SD. n = 78 and 82 for *AG1*^*II*^ and *AG1*^*ID*^, respectively). **G** Predicted protein structures of *PtoAG1*. The green helix structure, HD-ZIP domain; The red stem-loop, EAR motif; The yellow arrow, *AG1*^*In/Del*^. **H** Subcellular localization of PtoAG1 in *N. benthamiana* leaf cells. **I** Identification of PtoAG1 exhibiting transrepression activities by dual-luciferase reporter assay. The coding sequence of PtoAG1 was inserted into the Gal4BD vector to form PtoAG1-Gal4BD. These fusion vectors were co-transformed into *Populus* protoplasts along with firefly luciferase (LUC) and Renilla (REN) reporter vectors. VP16-Gal4BD and the empty Gal4BD vector were used as positive and negative controls, respectively. Data represent means ± SD from three biological replicates. Asterisks indicate significant differences as defined by Student's t-test (**P < 0.01, ***P < 0.001)

To dissect genetic tradeoffs between climate adaptation and growth in *Populus*, a pseudo-testcross mapping population was generated from '1236' × 'LM50' accessions, which exhibiting pronounced growth differentials across divergent bioclimatic zones (Additional file 1: Fig. S2A). Principal Component Analysis (PCA) of 11 collinear growth traits in the hybrid population revealed three principal components (PC1-PC3) capturing 93% of cumulative variance, serving as integrated phenotypic proxies (Additional file 1: Fig. S2B, C; Additional file 2: Tables S4,5). A high-density genetic map (87,562 SNPs) showed strong collinearity with the *P. tomentosa* reference genome (mean *ρ*-value = 0.95; Additional file 1: Fig. S3, Additional file 2: Table S6). QTL mapping identified qChr7.2 as a major locus for PC1 and three component growth traits (DBH, BSD and V; Fig. 2A; Additional file 2: Table S7), explaining 25.52–39.90% of phenotypic variance. Its colocalization with five GEA/GWAS signals governing adaptation and growth (Additional file 2: Table S3) prompted designation as qAG1 (QTL for ADAPTATION-GROWTH 1). Fine-mapping of qAG1 in another independent F_1_ population derived from hybridizing parental lines with pronounced growth differentials [34] (n = 550), delimited a 37-kb critical interval (SNP10-SNP14) via 14 recombinant events validated by PCR genotyping and progeny testing (Fig. 2B, C; Additional file 2: Table S8).

Among four annotated genes in this interval, *Chr7.86* exhibited significant spatiotemporal regulation, including suppressed expression during juvenile growth periods (Years 1–4) and vascular cambium-specific downregulation (Fig. 2E). CRISPR-Cas9 mutagenesis for the four candidate genes confirmed *Chr7.86* as growth regulators, with edited lines showing enhanced growth (Additional file 1: Fig. S4). Crucially, LM50 harbors a heterozygous frameshift mutation in *Chr7.86* (*AG1*^*ID*^, 305GA → G) causing premature translation termination (Fig. 2D), with no other functional variants detected in qAG1. Progeny inheriting *AG1*^*ID*^ exhibited superior growth versus *AG1*^*II*^ individuals (wild-type allele, Fig. 2F). We therefore designated *Chr7.86* as the causal gene underlying qAG1, hereafter named *PtoAG1*.

Association analysis of *PtoAG1* haplotypes across 302 accessions revealed that *AG1*^*In/Del*^ locus exhibited the strongest association signal. This locus stratifies the population into two spatially divergent ecotypes (Additional file 1: Fig. S5A, B), with *AG1*^*ID*^ accessions exhibiting significantly superior growth compared to *AG1*^*II*^ (*P* < 0.001) and predominating in Northeast (NE) and South (S) regions characterized by lower altitudes and higher precipitation seasonality (Additional file 1: Fig. S5C). Strikingly, *AG1*^*ID*^ aggregates in the Weihe Basin, coincident with the S subpopulation genetic cluster (Additional file 1: Fig. S5B and Fig. 1A). Allele frequency clines reveal strong geographical divergence: *AG1*^*ID*^ accounted for 40.8% in NW accessions but reached 94.6% in S and 100% in NE accessions (Additional file 1: Fig. S5C). Concurrently, selective sweep signals at the *PtoAG1* locus were detected in inter-subpopulation comparisons (Additional file 1: Fig. S5D). These results demonstrate that differential selection mediated by *PtoAG1* has driven local adaptation, sculpting allele frequency gradients along bioclimatic axes.

Protein sequencing confirmed *PtoAG1* encodes an HD-ZIP II transcription factor, containing conserved N-terminal tandem EAR repressors ([LxLxL]₂ motifs) and a C-terminal CPSCE environmental sensor (Additional file 1: Fig. S6). Structural modeling revealed EAR motifs adopt a stem-loop repression module, while HD-ZIP domain forms helix-turn-helix DNA-binding topology (Fig. 2G). Critically, the *AG1*^*ID*^ frameshift causes premature termination truncating the HD-ZIP domain (Fig. 2D), abolishing DNA-binding capacity. Phylogenetic analysis placed *PtoAG1* within HD-ZIP subgroup II, clustering with *Arabidopsis* orthologs *HAT9* (*AT2G22800*) and *HAT22* (*AT4G37790*) (Additional file 1: Fig. S7A). Subcellular localization confirmed PtoAG1 nuclear targeting (Fig. 2H). To systematically assess the transcriptional activity of *PtoAG1*, we first performed assays in the yeast strain Y2HGold. The results showed that yeast cells expressing the BD-*PtoAG1* construct, similar to the empty vector control, lacked transactivation activity (Additional file 1: Fig. S7B). To evaluate their activities in plants, we employed a transient expression assay in *Populus* protoplasts. The results demonstrated that the relative luciferase activity generated by *PtoAG1* was significantly lower than that of the empty vector control (Fig. 2I). These results suggest that *PtoAG1* function as repressors with transrepression activities.

### PtoAG1 constrains meristematic cell proliferation to regulate secondary growth

**Fig. 3:**
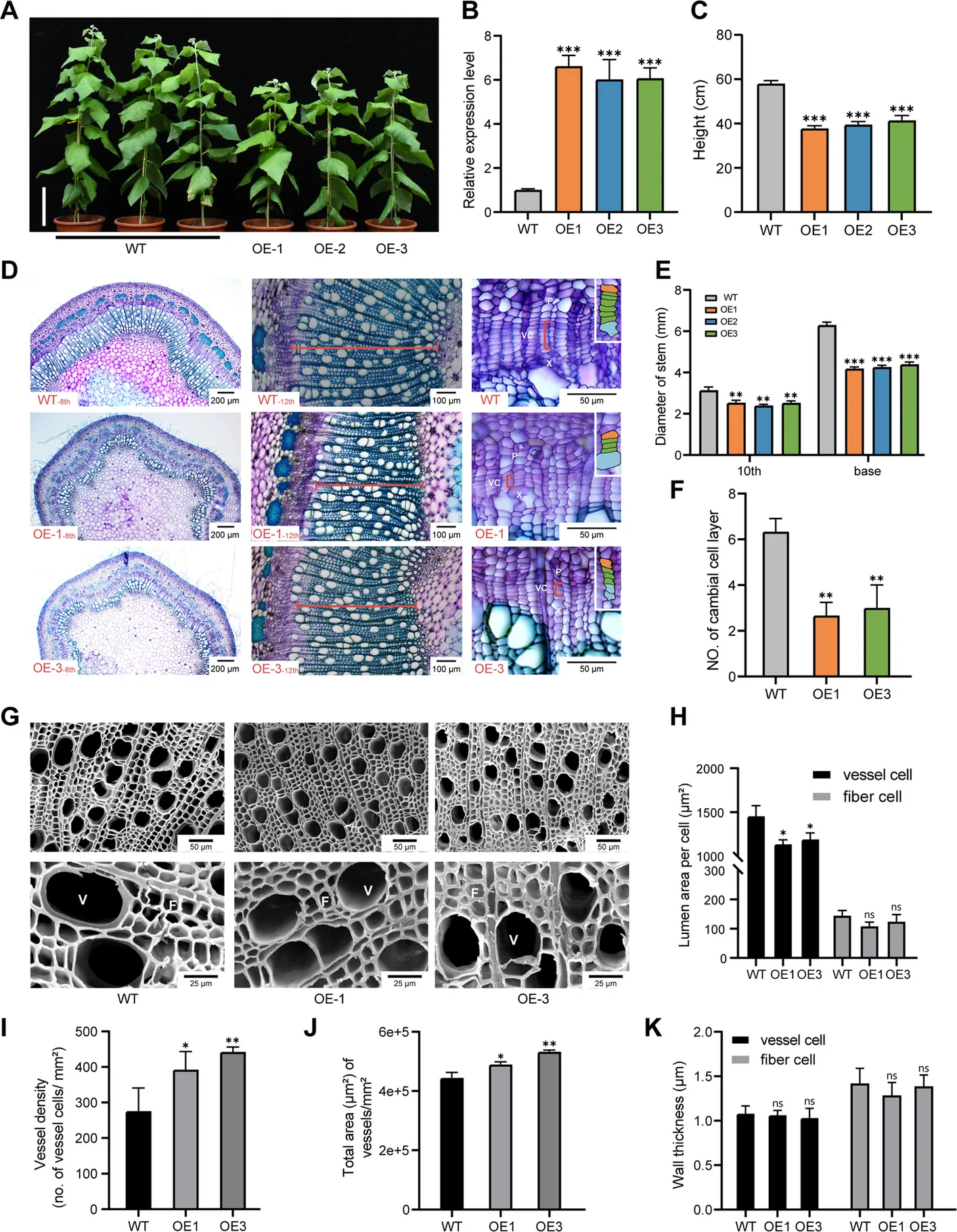
Characterizations of *PtoAG1* overexpression transgenic *P. alba* × *P. glandulosa*. **A** Phenotype of WT and *PtoAG1-OE* transgenic lines (OE-1, OE-2 and OE-3) 8wk after transfer to soil. Bar, 15 cm. **B** RT-qPCR analysis of *PtoAG1* in transgenic *Populus*. **C** and **E** Height and stem diameter of 2-mo-old plants. **D** Cytological observations of stem cross sections of 8th and 12th internodes with toludine blue. The red line in the middle figure represents the width of xylem. The insets of the rightmost picture show close-ups of cambium cells (green), adjacent phloem cells (orange) and adjacent xylem cells (blue). **F** Measurement of cambium cell layers of *PtoAG1-OE* transgenic poplar lines. **G** SEM of stem cross sections of 12th internode. **H–K** Measurement of lumen area (H), vessel density (**I**), cell wall thickness (**J**) and total area of vessels (**K**). In **B**, **C**, **E** and **F** error bars, SD (n > 5 biological replicates). In **H**–**K** error bars represent SD of 20 independent measurements. Two-tailed paired *t*-tests, *P < 0.05, **P < 0.01, ***P < 0.001. P, phloem; VC, vascular cambium; X, xylem; V, vessel; F, fiber cell

To further characterize *PtoAG1* function, we generated *Populus* transgenic lines overexpressing *PtoAG1* (*PtoAG1-OE*). All independent *PtoAG1-OE* plants exhibited significantly suppressed growth compared to wild-type (WT) (Fig. 3A-C; Additional file 1: Fig. S8A). Histological observation on stem cross-sections of 8th internode revealed that the *PtoAG1-OE* plants started to develop a continuous vascular cambium, and the interfascicular cambium showed only slightly differentiated compared to WT (Fig. 3D). WT displayed robust phloem caps and fully differentiated secondary xylem with continuous vessel-fiber circles, whereas *PtoAG1-OE* lines exhibited reduced xylem cell layers in both 8th and 12th internodes (Fig. 3D, E; Additional file 1: Fig. S8B, C), indicating xylem development impairment. To determine whether cambial activity alterations underlie this phenotype, we quantified vascular cambial zones. *PtoAG1-OE* lines possessed ~ 50% fewer cambial cell layers than WT (Fig. 3D, F). Scanning electron microscopy (SEM) corroborated these findings: vessel lumen area per cell decreased significantly in *PtoAG1-OE* lines, while vessel density and total vessel area increased (*P* < 0.05; Fig. 3G-J). Neither fiber lumen dimensions nor cell wall thickness differed significantly (Fig. 3H, K). These results demonstrate that *PtoAG1* overexpression inhibits cambial cell division, resulting in impaired secondary growth.

**Fig. 4:**
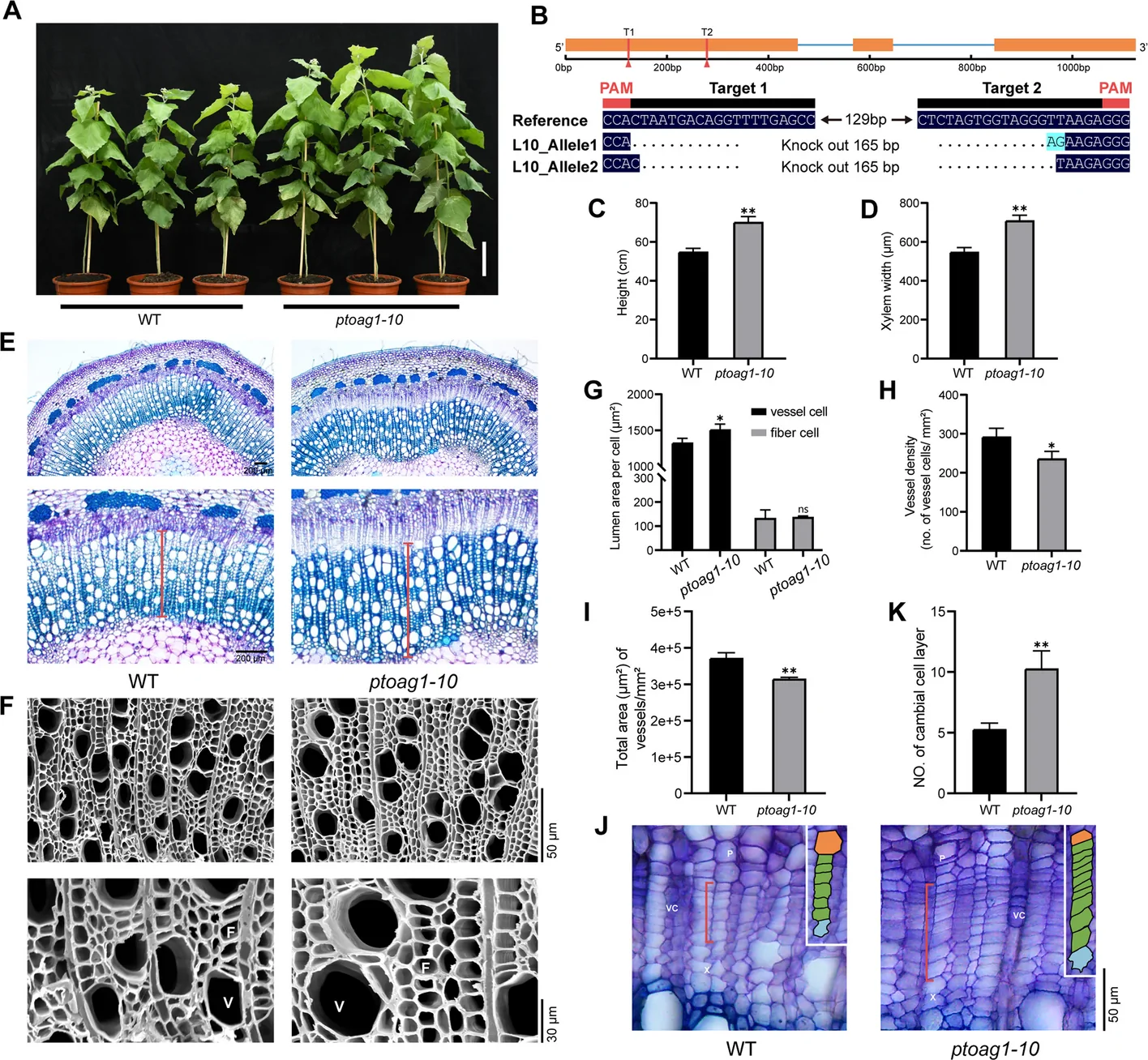
Characterizations of *PtoAG1* knockout mutants in *Populus*. **A** Phenotype of 8wk WT and *ptoag1-10* mutants generated through CRISPR/Cas9 genome editing. Bar, 15 cm. **B** Sequencing shows a 165 bp knockout between the target sites in the coding region of *PtoAG1* in the *ptoag1-10*. **C** and **D** Height and xylem width of 2-mo-old plants. **E** Cytological observations of stem cross sections of 10th internodes with toludine blue. The red line in the middle figure represents the width of xylem. F, SEM of stem cross sections of 10th internode. **G**-**I** Measurement of lumen area (**G**), vessel density (**H**), and total area of vessels (**I**). **J** Close-up of the cambium. The insets show close-ups of cambium cells (green), adjacent phloem cells (orange) and adjacent xylem cells (blue). **K** Number of cambial cell layers. Error bar represent SD values of 6 independent measurements (**C**, **D** and **K**) and 20 independent measurements (**G**, **H** and **I**). Two-tailed paired *t*-tests, *P < 0.05, **P < 0.01. P, phloem; VC, vascular cambium; X, xylem; V, vessel; F, fiber cell

To mechanistically dissect *PtoAG1* function, we engineered three CRISPR/Cas9 knockout lines in *Populus alba* × *P. glandulosa* [35] (Fig. 4A, Additional file 1: Fig. S9). Guided by natural variation patterns, sgRNAs targeted sequences between the EAR repression motifs and HD-ZIP DNA-binding domain (Fig. 4B). In contrast to PtoAG1 overexpression phenotypes, knockout mutants exhibited significantly enhanced growth versus WT: 27.9% increased height (*P* < 0.01; Fig. 4C), accelerated radial growth with expanded xylem zones (*P* < 0.05; Fig. 4D, E), 44% larger vessel lumen area (*P* < 0.05; SEM quantification, Fig. 4F, G), reduced vessel density yielding lower total conduit area (*P* < 0.05; Fig. 4H, I), and ~ 2.0-fold more cambial cell layers (*P* < 0.01; Fig. 4J, K). Enhanced periclinal divisions in knockout cambia drove xylem differentiation and radial expansion, confirming *PtoAG1* acts as a molecular brake in secondary growth. Crucially, overexpression of the natural variant *AG1*^*ID*^ in transgenic lines showed no detectable growth impact (Additional file 1: Fig. S10), confirming its functional null status. These results suggest *PtoAG1* as a negative regulator constraining meristematic cell proliferation.

### PtoAG1 reprograms xylem morphogenesis to confer drought resistance in Populus

**Fig. 5:**
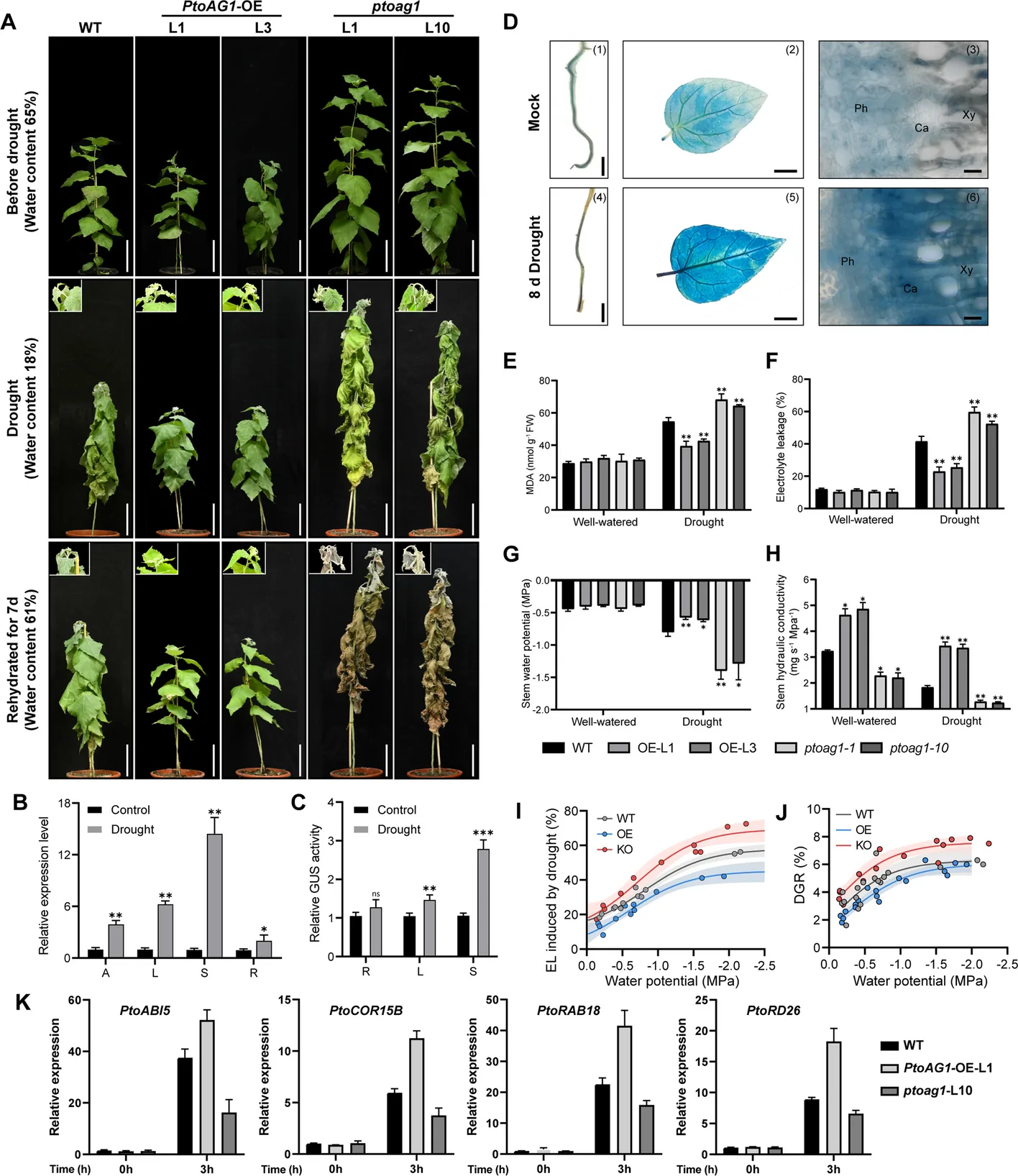
Morphology and physiology characteristics under drought stress. **A** The phenotype of plants under average soil relative water content (SRWC) 65%, 18% and rehydrated for 7d. Bars, 15 cm. The insets show close-ups of apical buds. **B** Relative *PtoAG1* expression levels of WT in different tissues. **C** Measurement of GUS activity driven by the *PtoAG1* promoter under mock and 8 d drought treatment. The values under mock treatment were normalized to 1. **D** GUS histochemical staining of the roots, leaves, and stem of 1-mo-old soil cultivated *AG1pro*::GUS transgenic lines under mock or 8 d drought treatment. Bars: (1, 4) 3 mm; (2, 5) 5 mm; (3, 6) 80 μm. **E**–**H** Determination of malondialdehyde (MDA) content (**E**), electrolyte leakage (**F**), stem water potential (**G**) and stem hydraulic conductivity (**H**) of plants. **I** and **J** Dynamics of electrolyte leakage (EL) and diameter growth rate (DGR) *vs* water potential under drought treatment for WT, *PtoAG1-OE*1, *ptoag1*−10. The dots represent individual measurements. The curves were obtained by fitting a sigmoidal model on the EL and DGR measured. Shaded areas are the confidence intervals of the models. Pseudo-*r*^2^ = 0.96, 0.94 and 0.97 for *ptoag1*, *PtoAG1-OE* and WT (**I**). Pseudo-*r*^2^ = 0.89, 0.92 and 0.66 for *ptoag1*, *PtoAG1-OE* and WT (**J**). K, qRT-PCR showed that the expression level of ABA-responsive genes*.* 1-mo-old plants were treated with 100 μM ABA for 0 h or 3 h. The relative expression levels of ABA-responsive genes in untreated WT were set to 1. Error bar represents SD values of 3 biological replicates. Two-tailed paired *t*-tests, *P < 0.05, **P < 0.01, ***P < 0.001. A, apical bud; L, leaf; S, stem; R, root

Building on histological evidence and GEA signals implicating *PtoAG1* in drought adaptation, we characterized its functional role under water deficit in *Populus*. Three-month-old WT, *ptoag1-10* mutants, and *PtoAG1-OE* transgenics were subjected to progressive drought until soil relative water content (RWC) reached 18%. At this threshold, WT plants showed leaf wilting and compromised apical buds, *ptoag1-10* mutants exhibited severe withering with apical bud collapse, while *PtoAG1-OE* plants maintained structural integrity (Fig. 5A). Following rehydration, *PtoAG1-OE* plants recovered rapidly, whereas the *ptoag1-10* mutants suffered near-complete mortality compared to WT. Drought significantly upregulated *PtoAG1* transcript levels in stems (RT-qPCR, *P* < 0.05; Fig. 5B). Tissue-specific induction was confirmed using *AG1pro*::GUS reporter lines, revealing strong drought-inducible GUS activity in stems (cambium and xylem) and leaves, but minimal response in roots (Fig. 5C, D). Crucially, size-matched plants (55 cm) confirmed that these drought resilience phenotypes were genotype-intrinsic (Additional file 1: Fig. S11), suggesting that the function of *PtoAG1* extends beyond its effects on stem growth and xylem structure to potentially modulate fundamental drought physiological response.

We quantified key physiological indices to characterize drought response dynamics across genotypes. Electrolyte leakage (EL) and malondialdehyde (MDA) levels—established biomarkers of stress-induced cellular damage [36], were significantly elevated in *ptoag1-10* mutants compared to WT. In contrast, *PtoAG1-OE* lines exhibited significantly lower levels of both markers (Fig. 5E, F). Hydraulic capacitance was assessed by measuring stem water potential (Ψ) [37] and hydraulic conductivity. Under drought stress, *PtoAG1-OE* maintained hydraulic conductivity (> 65% of baseline) and Ψ (> 80% retention), significantly mitigating osmotically-induced damage (Fig. 5G, H). These results support that the modified xylem anatomy induced by *PtoAG1* likely mitigates the actual degree of internal water deficit experienced by the plants under drought, suggesting that the differential physiological and biochemical responses (such as reduced oxidative damage) observed here may largely be secondary outcomes of these structural adaptations.

Time-resolved measurements integrating Ψ, EL, and diameter growth rate (DGR) revealed sigmoidal relationships [38]. Ψ was negatively correlated with both EL (pseudo-*r*^2^ = 0.96 [*ptoag1*], 0.94 [OE] and 0.97 [WT]) and DGR decline (pseudo-*r*^2^ = 0.89 [*ptoag1*], 0.92 [OE] and 0.66 [WT]) across genotypes (Fig. 5I, J). Consistent with these biomarkers, *ptoag1-10* mutants displayed sustained growth under moderate stress but experienced rapid mortality under extreme drought, indicating a failure in damage mitigation. *PtoAG1-OE* plants exhibited enhanced induction of drought-responsive genes upon treatment (Fig. 5K), provides indirect evidence of protective physiology against cellular damage.

We found that *PtoAG1-OE* lines possess a significantly reduced single-vessel lumen area (Fig. 3H, J), an adaptive xylem phenotype that reduces embolism risk [39]. Together with the concomitant physiological alterations observed in the transgenics, these findings collectively demonstrate that *PtoAG1* enhances drought tolerance by modulating xylem anatomy, which provides a structural basis that benefits the plant’s physiological status under severe stress.

### PtoAG1 represses PtoRR12 and PtoAREB3 to gatekeep CTK-ABA crosstalk

**Fig. 6:**
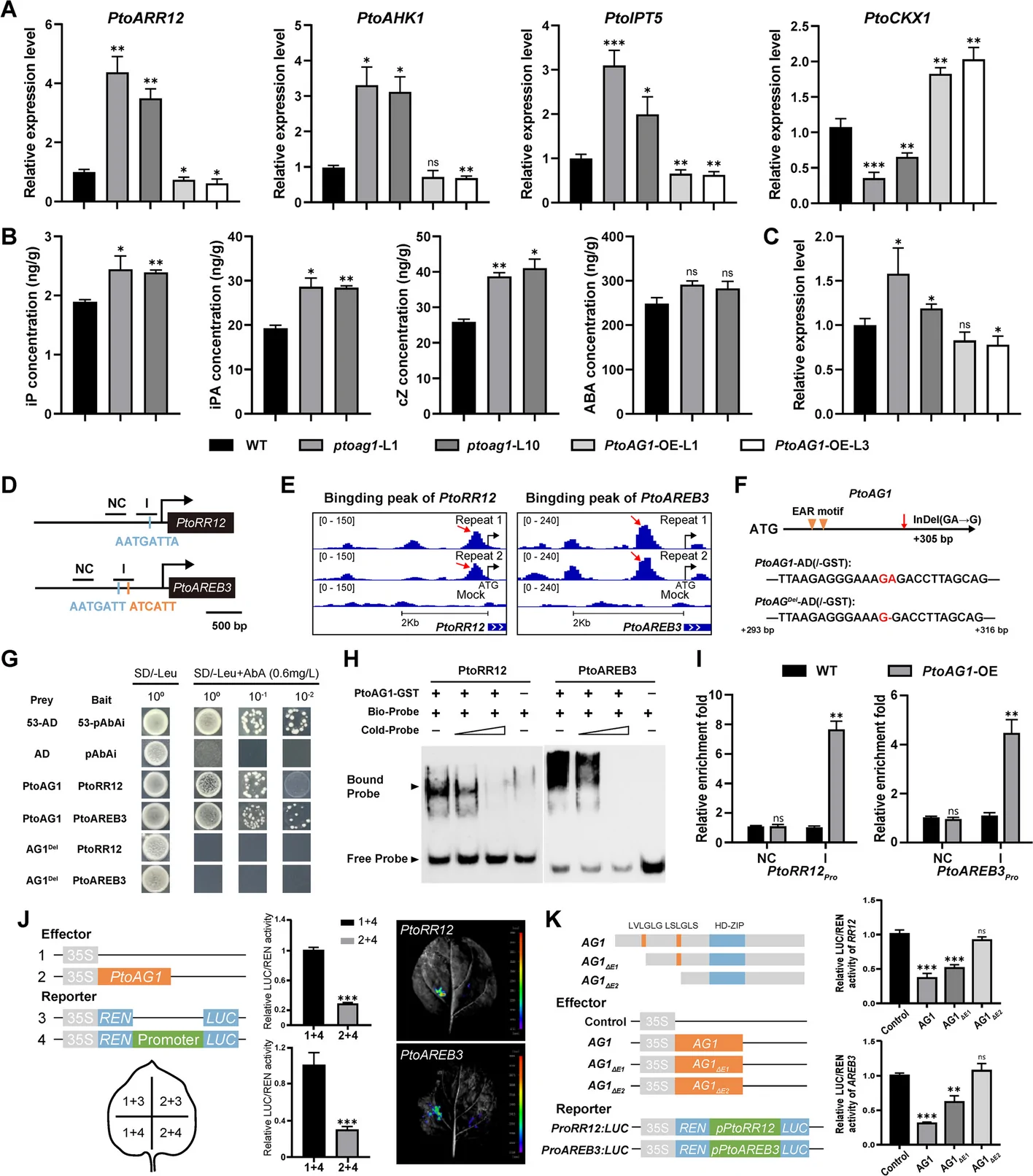
*PtoAG1* regulates the ABA and CTK signaling pathways. **A** qRT-PCR showed the expression levels of CTK-responsive genes obtained by DEGs. Data are presented as means SD from three technical replicates. **B** Quantitative analysis of cytokinin-related target metabolites. iP, N6-Isopentenyladenine; iPA, Isopentenyladenosine; cZ, cis-Zeatin. Data are presented as means SD from three technical replicates. **C** Expression levels of *PtoAREB3* in WT and transgenic lines. Error bars represent SD (*n* = 3 biological replicates). **D** Distribution of PtoAG1 binding sites (**I**) harbored in the promoter regions of *PtoRR12* and *PtoAREB3*. **E** Integrative genomics viewer images of the PtoAG1 DNA affinity purification sequencing (DAP-seq) read mapping to the promoters of *PtoAREB3* and *PtoRR12*. The red arrows indicate the binding peaks. **F** Sequence display of *PtoAG1* and the variation *AG1*^*Del*^ and the schematic representation of constructs used in Y1H and EMSA. **G** Y1H assays indicated that PtoAG1 can bind directly to the promoters of *PtoAREB3* and *PtoRR12*. *AG1*.^*Del*^ fails to bind to the promoters of *PtoAREB3* and *PtoRR12*. **H** EMSA of PtoAG1 binding to DNA probes of the *PtoAREB3* and *PtoRR12* promoters in vitro. **I** ChIP-qPCR analysis of the promoter region of *PtoRR12* and *PtoAREB3* enriched by PtoAG1 protein fused with GFP tag. NC, negative control; the short horizontal lines represent the location of the PCR-amplified fragments. (Three biological replicates were used in the ChIP assay.) **J** Left, a schematic of the transient co-expression of effectors and reporters. The full-length CDS of PtoAG1 was cloned into the pGreenⅡ 62-SK vector to serve as the effector, and the promoters of PtoAREB3 or PtoRR12 were cloned into the pGreenⅡ 0800-LUC vector to serve as the reporters. Middle, quantification was performed by normalizing firefly luciferase (LUC) activity to the activity of Renilla luciferase (REN). Right, representative images of luciferase activity. **K** Left, Schematic diagram of dual-LUC system of full length and truncated *PtoAG1* with EAR motifs deleted (ΔE1 and ΔE2). Right, quantitative results show that a reduction in EAR motifs leads to a weakened negative regulatory capacity of PtoAG1. Error bars are SD (*n* = 3 biological replicates). Two-tailed paired *t*-tests (*n* = 3), **P < 0.01, ***P < 0.001

To elucidate *PtoAG1*-dependent regulatory targets, we conducted transcriptomic profiling of WT and *ptoag1* mutants. Comparative analysis revealed 1,767 differentially expressed genes (DEGs; |log₂FC|≥ 1, *P* < 0.05) (Additional file 1: Fig. S12A). KEGG enrichment revealed significant overrepresentation (*P* < 0.05) of pathways governing carbohydrate metabolism, environmental adaptation, and signal transduction mechanisms (Additional file 1: Fig. S12B, Additional file 2: Table S9), consistent with observed phenotypic adaptations. Notably, CTK signaling components exhibited coordinated dysregulation: Positive regulators *PtoRR12* and *PtoAHK1* (two-component system) and CTK biosynthase *PtoIPT5* were significantly upregulated, whereas CTK catabolism gene *PtoCKX1* was downregulated (Fig. 6A andAdditional file 1: Fig. S12C). UHPLC quantification confirmed CTK metabolite accumulation: N⁶-Isopentenyladenine (iP): + 27.8%, Isopentenyladenosine (iPA): + 48.2%, and cis-Zeatin (cZ): + 53.9% (Fig. 6B). Concurrently, transcriptomic analysis revealed differential expression of genes within the ABA signaling network in *ptoag1-10*: the expression of the receptor PtoPYR1 decreased, while several negative regulators (PtoPP2C1/2/3/4) were upregulated (Additional file 1: Fig. S12D). Critically, these phosphatases (PP2Cs) inhibit SnRK2-mediated phosphorylation cascades [40], suggesting an altered response to ABA despite unchanged endogenous ABA levels in ptoag1 (Fig. 6B). The expression of the positive ABA signaling regulator PtoAREB3 was upregulated (Fig. 6C andAdditional file 1: Fig. S12D), suggesting the involvement of potential alternative regulatory pathways.

To identify direct transcriptional targets of *PtoAG1*, we performed DNA affinity purification sequencing (DAP-seq) to map its genome-wide binding sites (Additional file 1: Fig. S12E). KEGG pathway analysis revealed significant enrichment (*P* < 0.05) for phytohormone signaling pathways (Additional file 1: Fig. S12F). Integrated analysis of DAP-seq and transcriptome data nominated *PtoRR12* (CTK response) and *PtoAREB3* (ABA signaling) as top candidate targets (Fig. 6D, E; Additional file 2: Table S10). Moreover, homologs of these two genes displayed no significant expression changes in mutant DEGs (Additional file 2: Table S11). We validated these candidates through orthogonal approaches. Yeast one-hybrid (Y1H) and electrophoretic mobility shift assays (EMSA) confirmed direct binding of PtoAG1 to the promoters of *PtoRR12* and *PtoAREB3* (Fig. 6G, H). Chromatin immunoprecipitation (ChIP)-qPCR in *PtoAG1-OE* plants showed significant enrichment of target promoter fragments (Fig. 6I), demonstrating in vivo association. Consistent with these findings, dual-luciferase reporter assays (Dual-Luc) coupled with quantitative fluorescence measurements revealed that PtoAG1 represses transcriptional activity of both targets (Fig. 6J). To establish functional relevance, we generated transgenic overexpression lines for both target genes. *PtoRR12-OE* plants phenocopied *ptoag1-10* mutants, exhibiting enhanced stem growth but reduced drought tolerance (Additional file 1: Fig. S13A). Moreover, spatiotemporal transcriptome of cambial tissue demonstrated a negative correlation between *PtoAG1* and *PtoRR12* within the *PtoRR12* homologous gene family (Additional file 1: Fig. S13C). Conversely, *PtoAREB3-OE* lines recapitulated *PtoAG1-OE* phenotypes, showing growth retardation with increased drought resistance (Additional file 1: Fig. S13B). These results demonstrate that *PtoRR12* and *PtoAREB3* mediate distinct developmental and stress-responsive aspects of the pleiotropic effects of *PtoAG1*.

Functional dissection of PtoAG1 domains established the essential role of the HD-Zip domain in DNA binding. Y1H and EMSA assays confirmed that a truncated variant lacking this domain (PtoAG1^Del^, Fig. 6F) failed to bind promoters of *PtoRR12* and *PtoAREB3* (Fig. 6G and Additional file 1: Fig. S12G). Transient expression assays further demonstrated complete loss of transcriptional repression upon deletion of both EAR motifs, while partial truncation reduced repression efficacy by 35± 5% (*P* < 0.01; Fig. 6K). These results indicate that the naturally occurring *PtoAG1*^*Del*^ variant, through premature translation termination, abolishes repressor function by disrupting DNA-binding (via HD-Zip deletion), thereby preventing regulation of downstream target.

### PtoRR12 competitively inhibits SnRK2.2-AREB3 signaling to balance ABA-CTK crosstalk

**Fig. 7:**
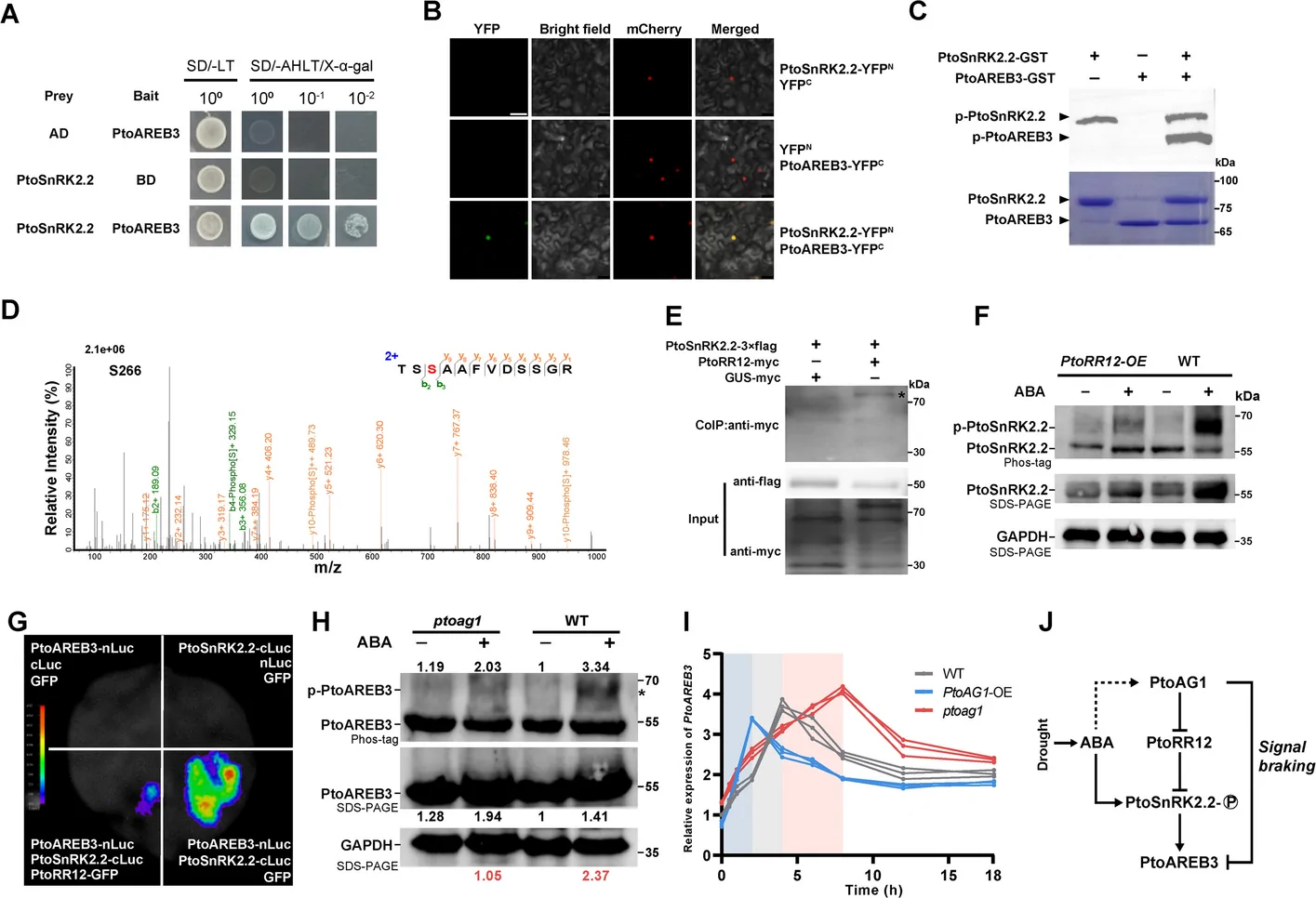
PtoSnRK2.2 phosphorylates PtoAREB3 and its auto-phosphorylation activity is inhibited by PtoRR12. **A** PtoSnRK2.2 interacts with PtoAREB3 in yeast cells. AD and BD represent empty vectors pGADT7 and pGBKT7, respectively. **B** Bimolecular fluorescence complementation assay (BiFC) showing interactions of PtoSnRK2.2 with PtoAREB3 in *Nicotiana benthamiana* leaf cells. Bars, 50 μm. **C** In vitro kinase assay of PtoAREB3 phosphorylation by PtoSnRK2.2. Coomassie brilliant blue (CBB) staining was used as a loading control (bottom). The exposure time was about 1 min. **D** Detection of phosphorylated amino acid residue in PtoAREB3 by LC–MS/MS. **E** Interactions between PtoSnRK2.2 and PtoRR12 analyzed using a CoIP assay. Flag-tagged PtoSnRK2.2 and myc-tagged PtoRR12 were co-overexpressed in *N. benthamiana* leaves. Anti-myc magnetic beads were used for immunoprecipitation. The asterisk indicates the bands of PtoRR12-myc. **F** Immunoblot analysis showing an upshift of PtoSnRK2.2 in phos-tag gels. Following 100 μM ABA treatment for 2 h, phosphorylated PtoSnRK2.2 protein levels were reduced in *PtoRR12-OE* lines. **G** Impact of protein interaction between the PtoRR12 and PtoSnRK2.2-PtoAREB3 complex. The in vivo interaction between PtoSnRK2.2-cLuc, PtoAREB3-nLuc and PtoRR12-GFP was tested via a LCI assay. **H** In-gel kinase assay showed PtoSnRK2.2 activity in WT and *ptoag1* lines after treatment with 100 μM ABA or mock for 2 h. The asterisk indicates the ABA-induced bands representing phosphorylation of PtoAREB3 by activated PtoSnRK2.2. The relative fold change in intensity of ABA-inducible bands in the mutants after ABA treatment was calculated and normalized to the WT controls. The red numbers represent the ratio of the phosphorylated protein band to the total protein band of PtoAREB3. I, Relative expression levels of *PtoAREB3* in WT, *ptoag1* and *PtoAG1-OE*. J, The braking regulatory pattern of *PtoAG1* on the PtoRR12-PtoSnRK2.2-PtoAREB3 cascade. All the experiments and measurements have been independently performed with three biological replicates and three technical replicates

The opposing drought-response phenotypes mediated by PtoRR12 (CTK signaling) and PtoAREB3 (ABA signaling) suggested inter-pathway crosstalk. Y1H and yeast two-hybrid (Y2H) assays confirmed no direct interaction between these effectors (Additional file 1: Fig. S14A, B). However, Y2H screening identified physical interactions between PtoRR12/PtoAREB3 and PtoSnRK2.2. We focused on a core kinase in the canonical ABA signaling cascade (SnRK2 → AREB/ABF) [41, 42]. Phylogenetic analysis identified three PtoSnRK2 homologs (Additional file 2: Table S12). Expression profiling revealed PtoSnRK2.2 as the most ABA/dehydration-responsive isoform (18.7-/9.5-fold induction; *P* < 0.001) (Additional file 1: Fig. S15A). Population-level transcriptomics (n = 50) confirmed coordinated induction of *PtoSnRK2.2* and *PtoAREB3* during drought and post-recovery dynamics (Additional file 1: Fig. S15B, C). We therefore focused on PtoSnRK2.2. Y2H and bimolecular fluorescence complementation (BiFC) assays confirmed it interacts with PtoAREB3 (Fig. 7A, B). In vitro kinase assays demonstrated robust phosphorylation of PtoAREB3 by PtoSnRK2.2 (Fig. 7C). LC–MS/MS analysis of the phosphorylated PtoAREB3 identified Ser266 as a major phosphorylation site (Fig. 7D). To validate the functional significance of this modification, we generated a phospho-null mutant (PtoAREB3^S266A^) by substituting Ser266 with alanine. Comparative phosphorylation assays revealed markedly reduced phosphorylation levels when using PtoAREB3^S266A^ as substrate compared to the wild-type protein (Additional file 1: Fig. S14C), establishing Ser266 as the primary phosphorylation target.

To define how PtoRR12 modulates ABA signaling, we systematically investigated its interaction with pathway components. Initial Y2H identified PtoSnRK2.2 as a PtoRR12-interacting partner (Additional file 1: Fig. S14D), a physical association subsequently confirmed in planta through BiFC and co-immunoprecipitation (Co-IP) assays (Additional file 1: Fig. S14E and Fig. 7E). Although in vitro kinase assays showed no detectable phosphorylation of PtoRR12 by PtoSnRK2.2 (Additional file 1: Fig. S14F), PtoRR12 suppressed PtoSnRK2.2 autophosphorylation. Consistent with this observation, in vivo phos-tag mobility shift assay under ABA treatment revealed a significant reduction in the abundance of phosphorylated PtoSnRK2.2 and PtoAREB3 in *PtoRR12-OE* plants compared to WT (Fig. 7F and Additional file 1: Fig. S14G), suggesting a potential regulatory role where PtoRR12 attenuates PtoSnRK2.2 kinase activity. Firefly luciferase complementation imaging (LCI) assays demonstrated that PtoRR12 competitively disrupts the PtoSnRK2.2-PtoAREB3 interaction in *Nicotiana benthamiana* leaves (Fig. 7G). To validate the rheostat function of *PtoAG1* in modulating ABA signaling activation and desensitization, phos-tag immunoblotting showed diminished ABA-induced PtoAREB3 phosphorylation in *ptoag1-10* mutants compared to WT (Fig. 7H), despite elevated PtoAREB3 protein levels. ABA treatment of transgenic lines revealed that PtoAREB3 is strongly induced by ABA, followed by the emergence of signal desensitization concurrent with increased *PtoAG1* transcription. Furthermore, *PtoAG1-OE* lines exhibited accelerated *PtoAREB3* response kinetics and attained regulatory homeostasis earlier (Fig. 7I, Additional file 1: Fig. S14H and I).

Collectively, our findings define a hierarchical regulatory module through which ABA-induced *PtoAG1* orchestrates drought response (Fig. 7J): *PtoAG1* transcriptionally represses *PtoRR12* to act as a fine-tuning amplifier, further enhancing the phosphorylation activity of PtoSnRK2.2, thereby promoting the activation of PtoAREB3; additionally, *PtoAG1* also directly represses the transcription of *PtoAREB3*.

### PtoSnRK2.2 phosphorylates PtoMYB84 to activate the PtoAG1-dependent ABA signaling feedback circuit

**Fig. 8:**
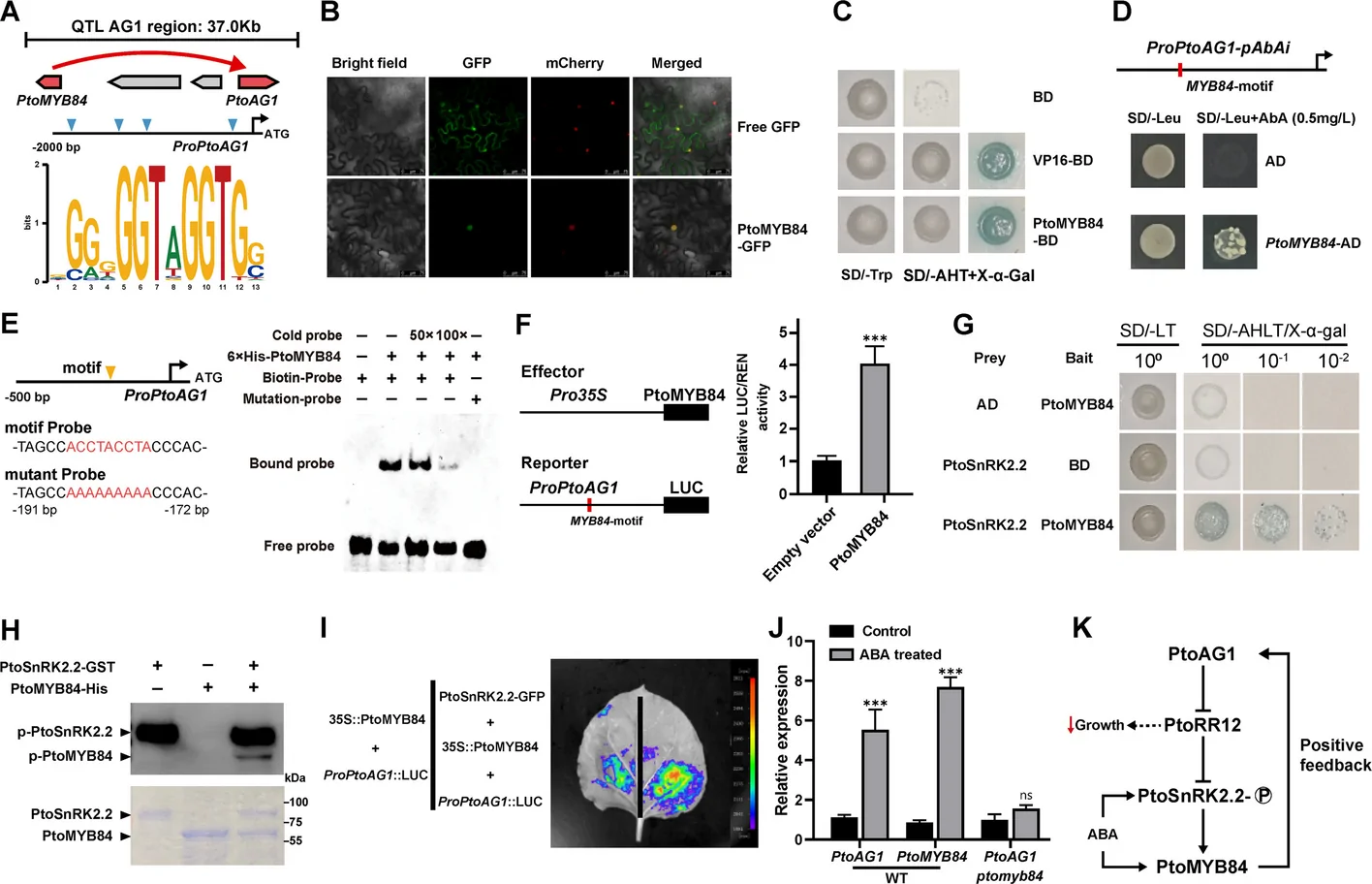
PtoSnRK2.2 phosphorylates cis-regulatory transcription factor PtoMYB84 acts upstream of *PtoAG1.* **A** Four PtoMYB84 binding sites were scanned in the promoter region of *PtoAG1*. Both *PtoMYB84* and *PtoAG1* are candidate genes for the significant QTL qAG1 in the fine mapping. The blue arrows indicate four *PtoMYB84* binding sites. The *P*-values of the motif scanning analysis are 3.18E-6, 2.92E-5, 3.52E-7, and 2.88E-5, respectively (from left to right). **B** Subcellular localization of PtoMYB84 in *N. benthamiana* leaf cells. **C** Transcriptional activation associated with PtoMYB84 in yeast cells. **D** Y1H assays indicated that PtoMYB84 can bind directly to the promoters of *PtoAG1*. **E** EMSA of PtoMYB84 binding to DNA probes of the *PtoAG1* promoter in vitro. The probe was biotin-labeled fragments of the *PtoAG1* promoter containing the PtoMYB84-motif, whereas the cold probe was unlabeled probe (50-, 100-fold molar excess). **F** A dual-luciferase assay indicated that PtoMYB84 activates the *PtoAG1* promoter in vivo. The promoter of *PtoAG1* was cloned into the pGreenⅡ0800-LUC vector, and the full-length CDS of *PtoMYB84* was cloned into the pGreenⅡ62-SK vector. Error bars represent SD of 3 biological replicates. Asterisks indicate significantly different values (***P < 0.001). **G** PtoSnRK2.2 interacts with PtoMYB84 in yeast cells. **H** In vitro kinase assay of PtoMYB84 phosphorylation by PtoSnRK2.2. Coomassie brilliant blue staining was used as a loading control (bottom). The exposure time was about 30 s. **I** Impact of protein interaction between the PtoSnRK2.2 and PtoMYB84-PtoAG1 complex. The in vivo interaction between PtoSnRK2.2-GFP, PtoMYB84-pGreenⅡ62-SK and PtoAG1-pGreenⅡ0800 was tested via a dual-LUC assay. **J** qRT-PCR showed that the expression pattern *of PtoAG1* and *PtoMYB84* in the WT or *ptomyb84-KO* lines under ABA treatment for 5 h. **K** The positive feedback activation regulatory mode of PtoMYB84 on the PtoAG1-PtoRR12-PtoSnRK2.2 cascade. All the experiments and measurements have been independently performed with three biological replicates and three technical replicates

CRISPR-Cas9-mediated knockout of four candidate genes within the *qAG1* QTL identified *Chr7.83* and *PtoAG1* loss-of-function mutants exhibiting significantly enhanced growth (1.3- and 1.4-fold height increase, respectively; *P* < 0.001; Additional file 1: Fig. S4). *Chr7.83* was annotated as an R2R3-MYB transcription factor, designated *PtoMYB84*. Promoter analysis of *PtoAG1* revealed four conserved PtoMYB84-binding motifs (core sequence GGTAGGTG [43, 44]; Fig. 8A, Additional file 2: Table S13). Subcellular localization analysis revealed that PtoMYB84 exclusively resides in the nucleus (Fig. 8B), and transcriptional activity assays demonstrated its function as a potent transcriptional activator (Fig. 8C). Y1H and EMSA assays established that PtoMYB84 specifically binds to the PtoAG1 promoter region (Fig. 8D and E). Dual-luciferase reporter assays confirmed this interaction, showing that PtoMYB84 significantly activates the PtoAG1 promoter (Fig. 8F), indicating that PtoMYB84 acts as an upstream transcriptional regulator of PtoAG1 expression.

To position the PtoMYB84-PtoAG1 module within ABA signaling, we found that PtoSnRK2.2 physically interacts with and phosphorylates PtoMYB84 (Fig. 8G, H). LC–MS/MS analysis further confirmed the absence of interactions between PtoSnRK2.2 and PtoAG1 (Additional file 2: Table S14). Co-expression of PtoSnRK2.2 with PtoMYB84 augmented *PtoAG1* promoter activation by 18.7-fold (*P* < 0.001 *vs.* PtoMYB84 alone; Fig. 8I), establishing a phosphorylation-dependent amplification loop. This regulatory triad exhibited coordinated ABA-responsive induction (9.2- and 5.2-fold for *PtoMYB84* and *PtoAG1*, respectively), whereas *ptomyb84* mutants showed attenuated *PtoAG1* expression under ABA treatment (Fig. 8J). Moreover, the *ptomyb84* mutants exhibited significantly compromised drought tolerance compared to the WT. Physiological assessments under drought stress confirmed this heightened sensitivity, as the mutants displayed a more severe physiological decline, characterized by accelerated reductions in stem RWC and stem water potential (Additional file 1: Fig. S16).

Thus, ABA activates PtoSnRK2.2 to enhance the PtoMYB84-mediated transcription of PtoAG1, which subsequently represses PtoRR12, a positive regulator of cytokinin signaling (Fig. 8K). Evolutionary analysis further revealed that this proximal PtoMYB84-PtoAG1 module suppresses recombinant segregation, underscoring its role in environmental adaptation.

### Evolutionarily conserved AG1 modulates growth-defense balance across plant lineages

**Fig. 9:**
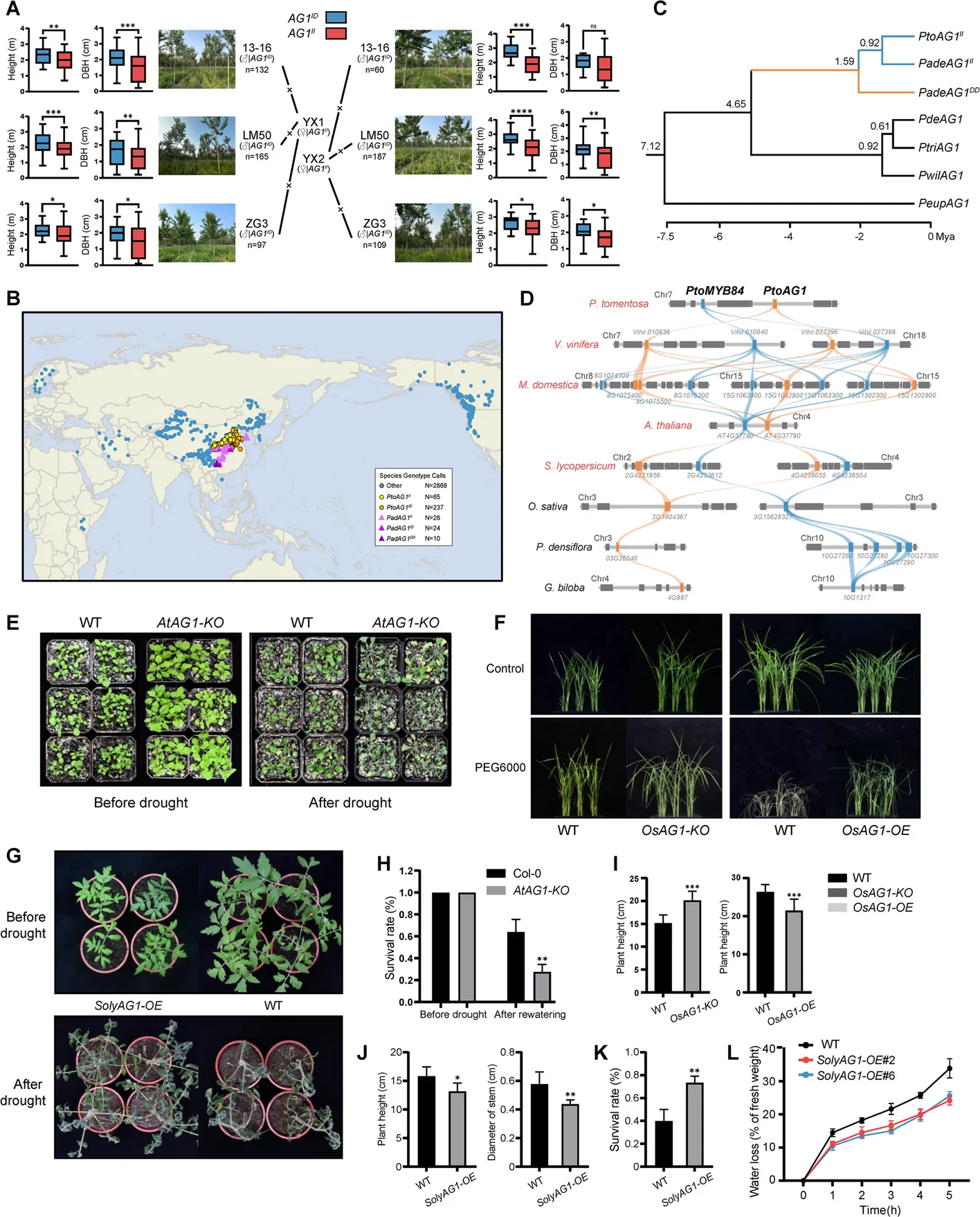
Breeding potential and evolutionary origins of *AG1.* **A** Plant height and diameter at breast height (DBH) of *AG1*^*II*^ and *AG1*.^*ID*^ individuals in six hybrid populations (n, the number of individuals in the hybrid families). **B** Genotyping of *AG1* in global georeferenced *Populus* species. **C** Phylogenetic analysis of *PtoAG1* coding sequence in six *Populus* species. Divergence times (Mya) are indicated in black numbers beside the branch nodes. **D** Syntenic relationships across *P. tomentosa*, *V. vinifera*, *M. domestica*, *A. thaliana*, *S. lycopersicum*, *O. sativa*, *P. densiflora, and G. biloba* genomes trace the ancient origins of the *AG1*-*MYB84* proximal regulatory module. Blue connections represent *MYB84* orthologs and orange connections represent *AG1* orthologs. Red species names denote species in which *MYB84* and *AG1* are a conserved module. **E** The phenotypes of Col-0 and *AtAG1-KO* lines before and after drought stress. **F** The phenotypes of rice WT, *OsAG1-KO*, and *OsAG1*-*OE* lines under hydroponic culture and PEG6000 osmotic stress. **G** The phenotypes of tomato WT and *SolyAG1-OE* lines before and after drought stress. **H** Survival rate of Col-0 and *AtAG1-KO* seedlings in soil before or after drought treatment. **I** Plant height of 14-day-old *OsAG1-KO* and 21-day-old *OsAG1-OE* seedlings relative to WT. **J** and **K** The plant height, stem width and post-drought survival rate of the tomato WT and *SolyAG1-OE* lines. **L** Water loss of the detached stem from 30-days-old WT and *SolyAG1-OE* lines, weighed at 1 h intervals. Error bar represents SD values of 3 biological replicates. Two-tailed paired *t*-tests (*n* > 5), *P < 0.05, **P < 0.01, ***P < 0.001

To quantify the breeding value of *PtoAG1*, we established six half-sib progeny populations comprising 750 F₁ hybrids derived from controlled crosses. Height growth in *AG1*^*ID*^ genotypes consistently exceeded *AG1*^*II*^ controls by 24.5 ± 3.1% (mean ± SD; *P* < 0.001), with a concomitant 30.3 ± 2.8% increase in stem diameter (*P* < 0.001) across all pedigrees (Fig. 9A). This transgenerational stability confirms *PtoAG1* as a robust selection target for *P. tomentosa* improvement.

Subsequently, to understand the origin of the natural variation *AG1*^*In/Del*^, analysis of 3,232 re-sequenced data from 57 species across five *Populus* sections revealed that the premature termination mutation in *AG1* was exclusive to *P. adenopoda* and *P. tomentosa* (Fig. 9B). Paleobotanical evidence indicates *P. adenopoda*—a thermophilic species endemic to subtropical China—expanded northward during the Mid-Pleistocene Transition (1.25–0.7 Ma), naturally hybridizing with *P. alba* to form *P. tomentosa* [45, 46]. The *AG1* allelic variant likely originated in *P. adenopoda* and introgressed into *P. tomentosa* via hybridization, evidenced by the high-frequency distribution (94.6%) of *AG1*^*ID*^ across southern China ecotypes proximal to the *P. adenopoda* native range. Time-calibrated phylogenies of *AG1* homologs from six section-representative species (Fig. 9C) confirmed the *AG1*^*In/Del*^ mutation arose 1.59 Ma (95% HPD: 1.72–1.46 Ma), with fixation in *P. adenopoda* during Early Pleistocene glacial cycles. Subsequent introgression into proto-*P. tomentosa* populations occurred ≈0.92 Ma (95% HPD: 1.01–0.84 Ma), coinciding with northward migration. Our findings delineate spatiotemporal trajectory of the key adaptive allele through hybridization-driven introgression.

Homology inference analysis within the *Populus* super-pangenome revealed that *AG1* and *MYB84* constitute a conserved co-regulated transcriptional module across five *Populus* species and *Salix purpurea*, with intergenic distances consistently constrained to < 40-kb (Additional file 1: Fig. S17). Phylogenetic extension to diverse species, including woody eudicots (*Vitis vinifera*, *Malus domestica*), herbaceous eudicots (*Arabidopsis thaliana*, *Solanum lycopersicum*), monocots (*Oryza sativa*), and gymnosperms (*Pinus densiflora*, *Ginkgo biloba*), confirmed strict conservation of this proximal module exclusively in eudicots (*P* < 0.001; Fig. 9D, Additional file 2: Table S15). Crucially, this genomic organization persisted through lineage-specific whole-genome duplications in Vitaceae, Rosaceae, and Solanaceae. These findings demonstrate that the *MYB84-AG1 cis*-regulatory architecture originated in ancestral crown eudicots ≈130 Ma and has been evolutionarily maintained to enable rapid environmental adaptation via growth-stress trade-off optimization [47].

To systematically evaluate the agricultural potential of *AG1*, we generated and characterized overexpression or knockout lines of its orthologs in three model crops: *Arabidopsis thaliana* (*AtAG1*), *Oryza sativa* (*OsAG1*), and *Solanum lycopersicum* (*SolyAG1*). Phenotypic analysis revealed that *AtAG1*-KO plants exhibited enhanced vegetative growth but reduced post-drought recovery by 61.0% relative to Col-0 (Fig. 9E, H). *OsAG1*-KO seedlings exhibited a 32.7% increase in plant height compared to WT (Fig. 9I), whereas *OsAG1*-OE lines conferred superior osmotic stress tolerance (Fig. 9F). Parallel observations in tomato demonstrated that *SolyAG1*-OE seedlings, while exhibiting slower growth rates (Fig. 9G,J), displayed dramatically improved drought survival rates (from ~ 40% in WT to ~ 73.3% in OE lines; Fig. 9K). Physiological measurements further revealed that detached stems from *SolyAG1*-OE plants retained water more efficiently, showing a 24.9% reduction in transpiration rate relative to WT controls (Fig. 9L). These cross-species analyses establish *AG1* as a key evolutionary modulator of the growth-defense balance. Its loss-of-function enhances vegetative growth, whereas overexpression prioritizes stress resilience—positioning *AG1* as a versatile genetic target for precision breeding in crops, from bioenergy feedstocks to climate-resilient cultivars.

## Discussion

As sessile organisms, plants evolved sophisticated plasticity to balance growth-stress tradeoffs. While ABA signaling universally orchestrates osmotic responses, its dynamic crosstalk with growth-promoting hormones remains poorly defined. Here, we identify the HD-Zip transcription factor *PtoAG1* (containing EAR motifs) as a master regulator that gates ABA sensitivity through CTK antagonism, coordinate xylem anatomy and drought responses for long-term adaptation in trees. Mechanistically: (i) Drought-induced PtoAG1 directly represses PtoRR12 (CTK signaling), relieving its auxiliary attenuation on PtoSnRK2.2 to amplify the primary ABA response; (ii) Activated PtoSnRK2.2 phosphorylates PtoMYB84, establishing a positive feedback loop via the PtoMYB84-PtoAG1 *cis*-regulatory module for rapid stress response amplification; (iii) Concurrent transcriptional repression of PtoAREB3 by PtoAG1 maintains signaling homeostasis. A natural frameshift mutation (*PtoAG1ᴰᵉˡ*) abolishes function but stably enhances hybrid growth across generations. Critically, *AG1* ortholog engineering in four plant models demonstrates conserved biotechnological utility: knockouts boost growth, while overexpression enhances drought tolerance.

HD-ZIP transcription factors regulate plant growth and local adaptation, representing prime targets for crop improvement [25, 26]. However, functional specialization among evolutionarily conserved HD-ZIP members remains poorly resolved. Previous studies implicated *Arabidopsis* HD-ZIP Ⅱ subfamily members in shade avoidance and shoot development [48, 49], with *hat3ahb4* double mutants showing abolished shoot apical meristem (SAM) activity and loss of WUS/CLV3 expression [50], suggesting that HD-ZIP Ⅱ members contribute to SAM initiation and stem cell maintenance. Through integrated GEA, GWAS, and fine-mapping, we identified *PtoAG1* as an HD-ZIP II gene governing growth-stress tradeoffs. Consistent with conserved meristem roles, both natural and CRISPR/Cas9-engineered *PtoAG1* loss-of-function alleles significantly enhanced meristematic activity (Figs. 4 and 9). Transcriptome profiling revealed concerted hyperactivation of CTK biosynthesis (*PtoIPTs*) and signaling (*PtoRRs*) genes (Additional file 1: Fig. S12), confirmed by UHPLC-quantified CTK metabolite accumulation (Fig. 6B). This CTK pathway hyperactivation aligns with established CTK signaling-biosynthesis feedback [51, 52], and may be potentiated by ABA-CTK antagonism [53].

Current studies establish that ABA signal transduction employs layered positive feedback loops, mirroring ABA biosynthesis pathways [54, 55]. This includes PYL receptor-mediated ABI5 induction, where ABI5 reciprocally sustains PYL expression [9], and SnRK2.3 phosphorylation of AREB1 activating sugar transporters (TST1/TST2), generating glucose efflux-triggered SnRK2.3 autoamplification [56]. These rapid signal escalation mechanisms enable swift stress responses [20, 57]. In our study, ABA/drought induction of *PtoAG1* occurs indirectly, without direct protein interaction with PtoSnRK2s, consistent with absent ABRE motifs in AG1 homolog promoters [28]. While PtoSnRK2.2-mediated phosphorylation provides the immediate acute stress response, the *PtoMYB84*-*PtoAG1* transcriptional loop functions as a primary regulatory driver for adaptation, rather than a secondary consequence of physiological decline. Induced within hours of stress (Fig. 8J) and sustainably expressed in the cambium, this module acts as a transcriptional brake. By transcriptionally repressing *PtoAREB3*, *PtoAG1* prevents runaway ABA signaling, executing a controlled desensitization strategy that is essential for maintaining signaling homeostasis and vascular survival over prolonged drought periods [10, 58].

**Fig. 10:**
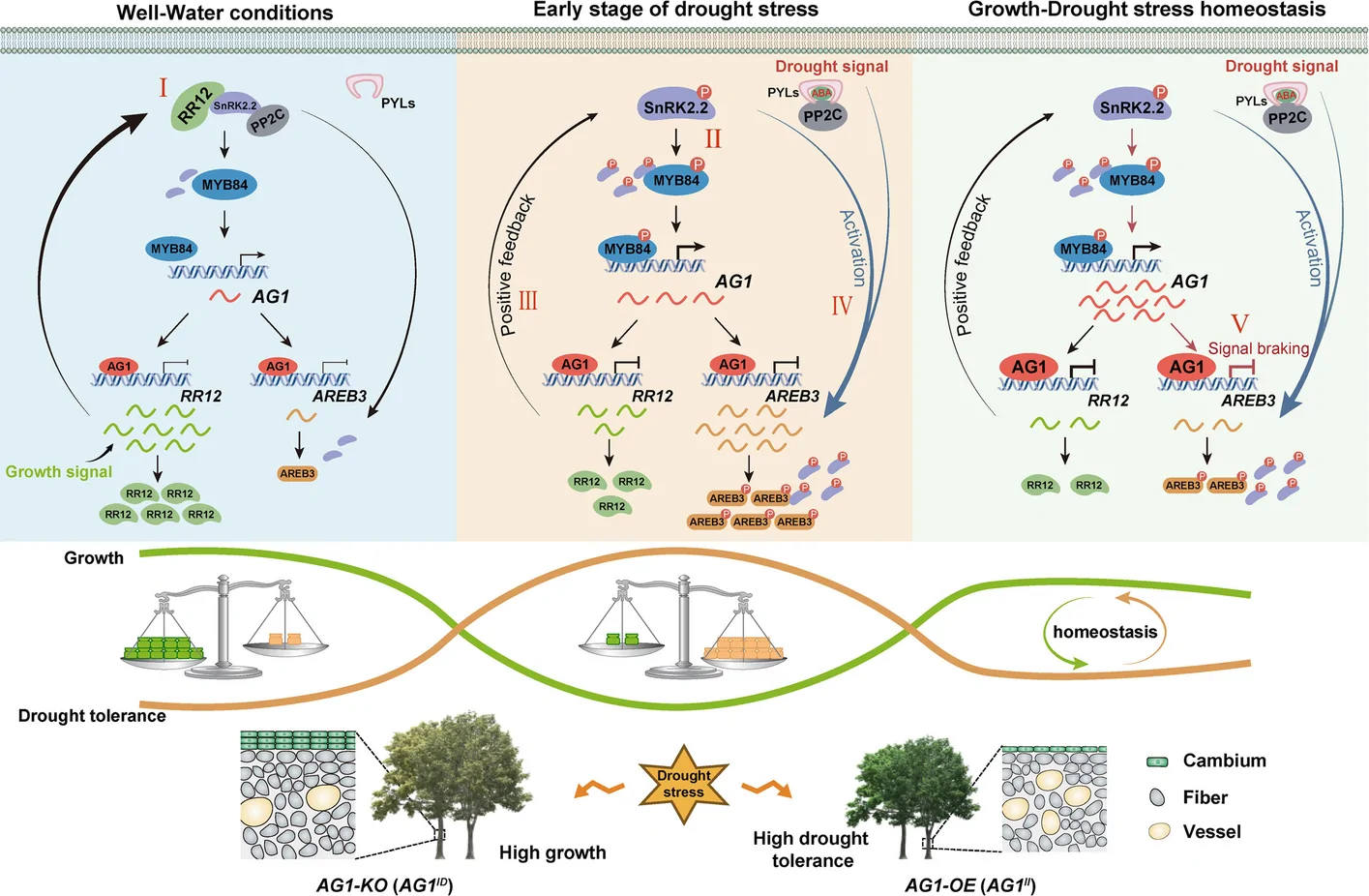
*PtoAG1* enables plants to rapidly respond to the transition between growth and drought tolerance through the “activation/braking” mechanism of ABA and CTK signals. Under normal conditions, the low expression of AG1 leaves *RR12* uninhibited. RR12 then transcriptionally activates downstream cytokinin-responsive genes, facilitating plant growth. Simultaneously, (I) RR12 inhibits the autophosphorylation of SnRK2.2. At this stage, the plants are in a state of high growth and low stress tolerance. Under stress conditions, ABA-bound PYR/PYL/RCAR receptors interact with and inhibit PP2Cs. (II) SnRK2.2 and MYB84 act as the first responders to promote the transcription of *AG1*. AG1 then significantly represses *RR12* to curtail growth. (III) The inhibition of RR12 releases the suppression on SnRK2.2 autophosphorylation, further facilitating *AG1* expression and thus forming a positive feedback loop in ABA signaling. (IV) SnRK2.2 then phosphorylates the ABA-induced AREB3, enhancing stress tolerance. (V) Finally, *AG1* represses the transcription of AREB3 to prevent ABA over-responsiveness, stabilizing the trade-off between plant growth and stress adaptation. Furthermore, *AG1* knockout lines display enhanced growth, increased cambial cell layers, reduced total vessel lumen area, and decreased drought tolerance—mirroring the natural *P. tomentosa* populations in humid southeastern regions. Conversely, *AG1* overexpression restricts growth, enlarges vessel lumen area, and improves drought tolerance, consistent with populations adapted to arid northwestern regions

Evolutionarily, the < 40-kb *MYB84*-*AG1* module is conserved across eudicots, positioning *MYB84* as the dedicated proximal activator of *AG1* (Fig. 9D). This gene cluster exemplifies a selection-stabilized co-adaptation model that prevents recombination-driven dissociation, ensuring unit inheritance. Functionally analogous to mammalian Hox clusters in coordinated transcriptional control [59], this cis-regulatory module likely occupies a shared topologically associated domain (TAD), enabling synchronized activation through stress-induced chromatin remodeling [60]. Thus, the *PtoMYB84-PtoAG1* feedforward loop is pivotal for rapid stress signal amplification and enhanced drought tolerance (Fig. 10).

Overactivated ABA signaling imposes significant growth penalties, exemplified by MdAREB1.1/1.2 autoregulation via ABRE-mediated self-amplification [56]. Thus, ABA pathways require precise desensitization mechanisms to balance stress resilience with developmental programs. While ABA-CTK antagonism coordinates these priorities [24] and CTK-response mutants exhibit ABA hypersensitivity [53], the molecular mediators remained undefined. Our findings reveal *PtoAG1* as a critical signaling integrator: (i) Growth modulation: ABA-induced PtoAG1 represses *PtoRR12*, suppressing cytokinin signaling to conserve energy during stress (Fig. 6J); (ii) Response amplification: The SnRK2.2 → PtoMYB84 → PtoAG1 cascade rapidly amplifies ABA responses (*PtoAREB3* activation; Fig. 8K); (iii) Attenuation circuit: PtoAG1 simultaneously represses *PtoAREB3* transcription (Fig. 6J), preventing ABA hyperactivation via transcriptional brake. This dual-regulation exhibits time-dependent potentiation under high ABA concentrations (Fig. 7G, I). Concurrently, the transcriptional dysregulation of PP2Cs likely represents a secondary negative feedback response downstream of the activated ABA cascade [58] (Additional file 1: Fig. S12D), as we confirmed the absence of any direct regulatory interaction between PtoAG1 and these PP2Cs (Additional file 1: Fig. S12H and I). Together with PtoRR12, these genes may act redundantly to ensure robust signaling desensitization, serving as an integrated mechanism to balance growth and stress responses [53, 61].

It is noteworthy that in *Arabidopsis*, homologs of *PtoAREB3* and *PtoABI5* are predominantly characterized by their roles in seed maturation and germination, whereas vegetative drought responses are largely governed by *ABF1*, *ABF2/AREB1*, and *ABF3* [62]. However, the recruitment of the “seed-type” effectors into the *PtoAG1* vegetative module in *Populus* likely reflects the distinct adaptive requirements of perennial woody plants. Following the Salicoid whole-genome duplication, extensive neofunctionalization occurred within transcription factor families to support long-term perennial growth [63]. Unlike annuals that escape adverse seasons as seeds, perennials must induce profound desiccation tolerance and dormancy within their mature vegetative tissues (e.g., buds and cambium) to survive environmental extremes. Consequently, seed-specific dormancy networks have been evolutionarily co-opted into vegetative stress responses in trees. Consistent with this, transcriptomic and functional studies in *Populus* have demonstrated that orthologs of *AREB3* and *ABI5* are robustly induced in vegetative tissues to govern bud dormancy, cambial activity, and drought adaptation [64, 65]. Thus, the prominent role of *PtoAREB3* in our drought-response module highlights an evolutionary rewiring of seed-associated ABA effectors to coordinate vegetative stress resilience in woody perennials. Furthermore, no functional compensation was observed among orthologs of the vegetative-dominant *AREB1/2* and *ABF3* (Additional file 2: Table S11). Collectively, *PtoAG1* represents a novel signaling integrator that fine-tunes the crosstalk between ABA and CTK signaling, establishing a tightly controlled homeostatic loop for ABA signaling (Fig. 10). This activating/braking mechanism enables plants to respond rapidly to abiotic stress, limiting energy expenditure in adverse environments. Furthermore, it alleviates the negative impact of ABA signaling on stem growth when the local environment returns to normal, facilitating a swift transition from a stress-tolerant state to a growth state. Thus, *PtoAG1* orchestrates ABA-CTK crosstalk through phased activation-desensitization cycles, optimizing fitness trade-offs under fluctuating environments.

Climate change threatensglobal biodiversity [66], necessitating identification of heritable adaptive variation for conservation prioritization [67, 68]. We demonstrate that natural allele *AG1*^*In/Del*^, a premature termination mutation, enhances vegetative growth and modulates local adaptation in *P. tomentosa*. Long-term environmental selection has driven climatic niche differentiation mediated by this allele, with loss-of-function genotypes enhancing vegetative growth while attenuating ABA sensitivity, thus dominating non-drought-prone southeastern China habitats (Additional file 1: Fig. S5B). These genotypes exhibit accelerated growth coupled with attenuated ABA sensitivity, aligning with *PtoAG1*’s role in hormone crosstalk. Crucially, breeding evaluations confirmed stable transgenerational inheritance of *AG1*^*In/Del*^ effects in hybrid crosses across *P. tomentosa* landraces (Fig. 9A). This establishes *AG1*^*In/Del*^ as a high-value target for precision breeding programs in this foundation species.

Strategic engineering of class II HD-ZIP transcription factors represents a promising approach for crop improvement, exemplified by the USDA-approved MON 874Ø3 maize line expressing a truncated ATHB17 variant that significantly enhances ear biomass (Monsanto Petition 14–213-01p). To address divergent breeding goals that yield maximization in favorable environments versus drought resilience in marginal areas, we modified *AG1* orthologs across model (*A. thaliana*) and crop (*O. sativa, S. lycopersicum*) species. We demonstrate that *AG1* knockout enhances growth while overexpression boosts drought tolerance, establishing a dual-intervention strategy for context-specific trait optimization. Notably, we identified *MYB84* as a conserved proximal enhancer of *AG1* that enables direct transcriptional regulation, bypassing spatial constraints of distal chromatin looping [69]. This evolutionarily conserved < 40-kb *MYB84-AG1 cis*-module in eudicots presents an attractive target for precision breeding using emerging genome-editing. The phylogenetic conservation of this module enables applications in staple crops (*Z. mays*, *S. tuberosum*) and perennial fruit species to address divergent agricultural challenges under climate volatility.

## Conclusions

Our study identifies the evolutionarily conserved *MYB84*-*AG1* cis-regulatory module as a critical molecular rheostat that fine-tunes abscisic acid and cytokinin crosstalk to orchestrate the plant growth-defense trade-off. By demonstrating how a natural frameshift mutation in *PtoAG1* drives geographic niche differentiation in *Populus*, we provide deep mechanistic insights into how woody perennials balance developmental and stress-response programs. Ultimately, these findings establish a comprehensive framework for plant environmental adaptation and highlight *AG1* as a highly translatable genetic target for the precision breeding of climate-resilient and high-yielding crops across diverse species.

## Methods

### Plant material and growth conditions

The germplasm population of *P. tomentosa*, consisting of 302 accessions, was randomly chosen from a clonal arboretum of 1,047 accessions in Guan Xian County, Shandong Province, China (36°23′N, 115°47′E), and asexually propagated via root cuttings in a randomized complete block design with three blocks in 2009 [70]. The population represented almost the entire natural distribution range of *P. tomentosa* (30–40°N, 105–125°E) and the collection sites of these 302 accessions were divided into the southern (S, *n* = 102), northwestern (NW, *n* = 92) and northeastern (NE, n = 108) geographical regions [71].

The full-sib family population 1 was established by crossing *P. tomentosa* ‘1236’ (female) and *P. tomentosa* ‘LM50’ (male), yielding 600 F_1_ hybrid individuals. 160 F_1_ individuals were randomly selected for constructing the mapping population and quantitative trait loci (QTL) analysis. The progenitors and F_1_ individuals were grown in Guan Xian County in 2018, using a randomized complete block design with three replications. The population 2 consisting of 550 individuals were described in detail previously [34].

*P. alba* × *P. glandulosa* “84 K” were used as the biological basis for genetic transformation. The sterile plants were propagated through cuttings on woody plant medium (WPM) under a long-day photoperiod (24 ℃, 16 h of light/8 h of dark). Then, 30-day-old asexually propagated poplars were transplanted into soil and maintained in a greenhouse with the same photoperiod conditions. Arabidopsis (*A. thaliana*) ecotype Columbia-0 was used as WT. Seeds were sterilized and cold-stratified at 4 ℃ for 4 days and then grown on Murashige and Skoog (MS) medium in a growth room at 22 ℃ with a 16/8 h photoperiod. The rice (*Oryza sativa* L.) plants used in this study were in the *japonica* variety ZH11 background. Rice seedlings were hydroponically cultivated in a chamber with a 16 h light (28 ℃)/8 h dark (25 ℃) regime and the nutrient solution (Kimura B) was renewed every 2 d. Tomato (*S. lycopersicum*) cultivar Ailsa Craig was used as WT background.

### Vector construction and genetic transformation

To obtain the various knockout lines of 4 genes within the QTL qAG1 *PtoMYB84*/*Chr7.84*/*Chr7.85*/*PtoAG1*, *PtoSnRK2.2/2.3* double mutants, the target sequences were designed via CRISPR-direct (http://crispr.dbcls.jp) and the sgRNA expression cassettes targeting these genes were introduced into the pYLCRISPR/Cas9-DN vector [72]. To generate the *PtoAG1*-, *PtoRR12*-, *PtoAREB3*-overexpression lines, the full-length cDNAs of each gene were amplified with specific primers. The PCR products were cloned into pBI121 vector by using the Uniclone One Step Seamless Cloning Kit (Genesand Biotech). To construct the *AG1pro*::GUS vector, 1500 bp fragment upstream of *PtoAG1* start codon was amplified and assembled into pCAMBIA2300-MCS-GUS vector. All transgenic plants were generated via *Agrobacterium* (GV3101)-mediated transformation as previously described [73].

To obtain the *AG1* mutants of Arabidopsis and rice, the same genome-editing system (as described above) was employed to construct the *atag1* and *osag1* mutants by BIOGLE GENE TECH company (http://www.biogle.cn). For *SlAG1-OE* lines, CDS for *SlAG1* was PCR amplified from tomato cDNA and cloned into the pBI121-GUS vector. Primers for vector construction and the RT-qPCR assay are listed in Additional file 2: Table S16.

### ABA, fluridone, PEG6000, and drought stress treatments

One-month-old poplar were treated in liquid MS medium with 100 μM ABA or 50 μM fluridone for the specified time. Three individual plants after treatment were harvested and stored at −80 ℃ for further analysis. For the osmotic stress tolerance assay, the poplar or rice seedlings were treated with 20% PEG6000 for 3-7d.

For moisture-controlled drought treatment, 3-month-old or 55 cm tall poplars underwent drought stress, in which the soil relative water content (RWC) was reduced from 65% (before drought) to 18% or 22% (after drought) by holding water, and rehydrated for 7d. One-month-old tomato plants were subjected to drought stress by the same method. The SRWC was detected with a soil moisture detector (ML3; Delta-T Devices Ltd., Cambridge, UK). Stems of mock and drought-treatment poplars were harvested for physiological indicators measurement. The malondialdehyde were measured using a detection kit (Solarbio, Beijing, China), according to the manufacturer’s instructions. Electrolyte leakage measurement was used to assess the cell damage in stems induced by drought. Three stem samples per individual were collected. Samples were cut into 3 cm–long sections and immersed in vials containing 15 ml of pure water. Stem water potential was measured using A SAPS II Water Potential System (Zhejiang Top Cloud), according to the manufacturer’s instructions [107]. Stem hydraulic conductivity was measured by the high-pressure flow meter technology.

### Phenotypic evaluation

For genotype-environment association, 19 BIOCLIM environmental variables from WorldClim [74] (Additional file 2: Table S1) in the provenance regions of the germplasm population of *P. tomentosa* were used for the analysis. In the further redundancy analysis (RDA), the 19 environmental variables were considered the ranked importance and the distribution of genetic diversity by using GF analyses with R package “gradientForest” [75] and correlations (pairwise correlation coefficient |*r*|< 0.8). For the dynamic stem growth of GWAS, the height and diameter-at-breast height were measured at eight-time pints of 302 *P. tomentosa* accessions during December in 2011 to 2018, with at least three replicates for each measurement. This time span corresponds to the 3rd to 10th year of tree growth [32]. For the growth traits of QTL mapping, a total of 4 biomass traits (plant height [H], diameter at breast height [DBH], diameter of stem basal [SBD], and stem volume [V]), 3 photosynthetic traits (the net photosynthetic rate [A], transpiration rate [E], and global conductance to CO_2_ [GTC]) and 4 leaf shape traits (leaf length [LL], leaf width [LW], leaf area [LA] and leaf stalk length [LS]) were scored for each accession, with at least three replications per genotype. All traits were scored using standard procedures described previously [76, 77]. To comprehensively evaluate tree growth and reduce phenotypic dimensionality, principal component analysis (PCA) was performed to extract key information from 11 phenotypically complex traits that are highly correlated with plant growth. The first 3 principal component (PC) were retained for QTL mapping.

### Genomic resequencing and SNP calling

The methods and clean data for resequencing the association population with 302 accessions and the mapping population were previously described [78]. In brief, clean reads were aligned to the *P. tomentosa* reference genome by the Burrows-Wheeler Aligner (v0.7.17-r1188) using default parameters [79]. The low mapping quality (MQ < 20) reads were filtered using SAMtools [80] (v1.12) and the marked-duplications were removed by Sambamba_v0.6.6 [81]. Genome variants calling was performed using GATK v.4.1.5 (https://gatk.broadinstitute.org/hc/en-us) with the official hard filtering parameters. VCFtools_0.1.13 was used to extract high-quality SNPs with two alleles, a minor allele frequency (MAF) > 0.05, a maximum missing genotype < 0.2.

### RNA-seq and data analysis and quantitative real-time PCR (RT-qPCR) analysis

Total RNA was extracted from *P. tomentosa* plants following the operating instruction of the FastPure Plant Total RNA Isolation Kit (Vazyme, Nanjing, China). First-strand cDNA was synthesized by using the Reverse Transcription System (Promega Corporation, Madison, WI). Real-time quantitative polymerase chain reaction (RT-qPCR) was conducted using SYBR Premix Ex Taq (TaKaRa, Dalian, China) on a QuantStudio 6 Flex Real-Time PCR System (Thermo Fisher Scientific, Waltham, MA). All reactions were performed with three technical and three biological replicates. The *P. tomentosa* Actin gene was used as the internal control to normalize the gene expression level. A list of primers used for RT-qPCR is given in Supplementary Table 16. For the transcriptome of different tissues, a number of tissue types, including shoot apices, leaves, phloem, cambium, developing xylem, and mature xylem of 1-, 2-, 3-, 4-, 5- and 10-year-old *P. tomentosa* clones ‘LM50’ planted in Guan Xian County, were subjected to RNA-seq analysis [108]. For the population-level transcriptome of drought and post-recovery dynamics, one-year-old *P. tomentosa* individuals, which were clonally propagated via root cuttings in Guan Xian County in 2018, were subjected to a 30-day drought treatment followed by a 20-day rewatering period. For the transgenic poplar, the stem tissues of the tenth internodes of 3-month-old transgenic poplar, together with those of WT, were used for RNA-seq. Total RNA was extracted using the Qiagen RNAeasy kit (Qiagen China, Shanghai, China) with three biological replicates. Paired-end sequencing was performed on an Illumina HiSeq 2000 system (Illumina, San Diego, CA). The acquired clean data were mapped to the “Tianshui” genome using HISAT2 [109]. The BAM files for all the RNA-seq samples were assembled using Stringtie v.2.0.4 [110]. Hierarchical clustering was performed by the MeV software https://sourceforge.net/projects/mev-tm4/files/ with the HCL method. DEGs were identified using DEseq2 [111].

### UHPLC-MS/MS analysis

Stem materials used for RNA-Seq were also used for UHPLC-MS/MS analysis. The types and contents of phytohormone target metabolites in the stems of *P. tomentosa* were detected using ultra-high-performance liquid chromatography-tandem mass spectrometry (UHPLC-MS/MS) by BioMarker Technologies (Beijing, China). Stem materials used for RNA-Seq were also used for UHPLC-MS/MS analysis. The types and contents of phytohormone target metabolites in the stems of *P. tomentosa* were detected using ultra-high-performance liquid chromatography-tandem mass spectrometry (UHPLC-MS/MS) by BioMarker Technologies (Beijing, China). Briefly, an appropriate amount of sample was weighed into a 2 mL centrifuge tube, followed by the addition of a steel bead and 1 mL of isopropanol-water solution, and the mixture was vortexed for 30 s. The sample was homogenized at 40 Hz for 4 min and then centrifuged at 14,000 r/min for 15 min. A total of 800 μL of the supernatant was collected and concentrated to dryness by rotary evaporation. The residue was reconstituted with 160 μL of methanol–water solution (50:50, v/v). After centrifugation at 14,000 r/min for 10 min, the supernatant was passed through a 0.22 μm filter membrane prior to injection for analysis. Data acquisition was performed using an ultra-performance liquid chromatograph (Waters ACQUITY I-Class; Waters Corporation, Milford, CT, USA) and a tandem mass spectrometer (SCIEX QTRAP 6500 +; SCIEX, Marlborough, MA, USA). Metabolite quantification was conducted using the scheduled multiple-reaction monitoring method [112].

### GWAS strategy and population genomic analyses

Two environment-variation association models, namely a univariate latent-factor linear mixed model (LFMM) and redundancy analysis (RDA), were employed to identify adaptive variations across the entire genome in the natural population of 302 *P. tomentosa* individuals. Within the same population, a mixed linear model was used to detect significant variations associated with dynamic growth phenotypes of 8-yr. GWAS was conducted with a total of 12,672,386 high confidence SNPs with minor allele frequency (MAF) > 0.05 and missing rate < 0.2. SNPs were filtered using PLINK v1.90 with the parameters "indep-pairwise 50 10 0.2" to generate a LD-pruned dataset. Population structure was inferred by Admixture v1.3.0 software [113] with the cluster number K from 1 to 10. To avoid parameter standard errors, each K was run with ten replicates. K = 3 was chosen based on the minimum cross-validation (CV) error value. For the environment-variation association, we firstly used a LFMM model implemented in the R package LEA v3.3.2 [114] to identify for associations between allele frequencies and the 19 BIOCLIM environmental variables. Second, RDA implemented in the R package vegan was employed to detect genetic variants strongly associated with multivariate environmental axes. The relative importance of 19 bioclimatic variables was ranked using the gradient forest model from the R package gradientForest [115]. Subsequently, Pearson correlation analysis was used to select five climate variables (Bio4, Bio6, Bio8, Bio13, and Bio17) with pairwise correlation coefficients |r|< 0.8 for inclusion in the RDA. For the dynamic growth associations, EMMAX was utilized to generate a kinship matrix for all samples and test for associations between SNPs and all traits [116]. To address this issue, the Genetic type 1 Error Calculator (GEC) software was employed to estimate the effective number of independent markers (n), yielding a suggested threshold of 4.88 × 10–7 (1/n, where n = 2,048,622) for controlling the type I error rate. Genes located within 2 kb upstream or downstream of GWAS signals were considered candidate genes. To search for genomic region of selection sweeps, we used a 10-kb sliding-window with a step size of 5 kb to calculate the nucleotide diversity level (π) and Fst between pairs of three geographical populations (NW, NE, and S). If π for a window was less than 0.001 in a population, the window was excluded. Population differentiation was evaluated using the fixation index (FST). Pairwise FST values among the three populations were calculated using a sliding window approach (10 kb windows sliding in 5 kb steps) implemented in VCFtools [117]. Genome regions within the top 5% of both the π ratio and FST distributions were designated as candidate sweep regions. Additionally, the summary statistic Tajima’s D was computed using VCFtools to identify signatures of selective sweeps within genomic regions. To investigate the roles of geographic and environmental factors in shaping adaptive (546 adaptive variants identified by LFMM and RDA) and neutral (718,114 LD-pruned SNPs as used for population structure analyses) variations during the formation of spatial genetic differentiation, Mantel tests and partial Mantel tests were used to analyze the associations between* F*_*ST*_/(1-*F*_*ST*_) and both isolation by distance (IBD) and isolation by environment (IBE) with significance determined using 999 permutations in the R package *vegan* [82]. Geographic distances were calculated using the R package *geosphere* [83]. For environmental distances, climate variables were first scaled and centered for numerical standardization, and pairwise Euclidean distance matrices were then computed using the R package *vegan*. Geographic distribution maps of *Populus* spp. individuals across China or globally were generated using ArcGIS 10.2.

### Construction of genetic map, QTL mapping, and fine-mapping

To maximize the filter for the false-positive and uninformative SNPs, we used GATK to remove the clusters of variants within a 10 bp window region with 3 or more SNPs [118]. The SNPs coded as aa × bb and markers that do not conform to genetic laws were removed by the Onemap package in R [119], and the other bi-parental markers of segregation patterns (ab × cd, ab × cc, cc × ab, ef × eg, hk × hk, lm × ll and nn × np) were considered suitable for the cross-pollinator population type. Then the polymorphic SNP markers showing significant segregation distortion (the allelic separation ratio is significantly deviated from the expected mendelian ratio) according to a two-sided χ2 (chi-squared) test were excluded from further analysis (*P* < 0.01). In total, 93,204 high-quality SNPs were obtained and the original genetic map was then constructed by Lep-MAP3 v.0.5 [120]. The construction of bin map was performed using Onemap, and the deletion of redundant markers was based on genetic distances obtained with the original framework map [121]. The LinkageMapView package and ALLMAPS were separately used to visualize the genetic map and perform the collinear analysis with default parameters [84, 85]. QTL analysis of 11 growth traits and 3 comprehensive traits (PCs) was performed using the inclusive composite interval mapping (ICIM) method with GACD v1.2 software. LOD threshold values for evaluating the statistical significance (0.05 significance level of Type I error) were estimated by 1000 permutations to determine the significant QTLs. The other parameters were set to defaults. The same significant QTLs detected in both the single growth trait and the comprehensive trait were defined as the stable QTLs. The mapping populations consisting of 160 (Population 1) and 550 (Population 2) individuals were planted in 2018 and 2008 in Guan Xian County, respectively. To fine map the qAG1 locus, we developed new molecular markers (SNPs and InDels) for genotyping in the region containing *PtoAG1* according to the “Tianshui” reference genome. Crossovers were mapped in recombinant F1 individuals by genotyping the SNP markers indicated in Supplemental Table 8, which were developed and genotyped through PCR amplification and Sanger capillary electrophoresis sequencing.

### Dual-luciferase assay

The full-length sequences of *PtoAG1* and *PtoMYB84* or partial truncation of *PtoAG1* were inserted into the pGreenⅡ62-SK vector as effectors. To construct the reporter, the promoters of the target genes were cloned and inserted into the pGreenⅡ0800-LUC vector. Equal volumes of Agrobacterium cells which were transformed with constructs were co-transformed transiently into *N. benthamiana* leaves. After 48 to 60 h of infiltration, leaves were sprayed with a 1 mM D-fluorescein and incubated in the dark for 3 min before capturing images. Quantification was performed using a Dual Luciferase Assay kit (E1910, Promega, USA) according to the manufacturer’s instructions.

### Subcellular localization and transcription activation assays

The full-length coding sequence of *PtoAG1* and *PtoMYB84* was amplified by PCR and recombined with the *pBI121* vector, which contains the *35S* promoter and GFP tag. The construct was introduced into *A. tumefaciens* strain GV3101, which was then used to infiltrate leaves of *N. benthamiana*. Two days post-infiltration, observations were conducted using a Leica SP8 confocal laser scanning microscope (Leica Biosystems, Nussloch GmbH, Germany). Each experiment included three biological replicates.

The trans-activation activity assays were performed as previously described [86]. Briefly, the CDSs of PtoAG1 and PtoMYB84 were fused with the VP16 fragment and inserted into the pGBKT7 (Clontech, Mountain View, CA) vector, and the resulting recombinant plasmids, the positive (VP16-BD), and negative (BD) controls were introduced into the Y2H yeast strain. Positive transformants were selected on synthetic dextrose medium lacking tryptophan (SD/-Trp). These transformants were subsequently inoculated onto SD medium deficient in tryptophan, histidine, and adenine (SD/-AHT) for transactivation assays. X-α-gal was used to assess the transcriptional activation activity of *PtoAG1* and *PtoMYB84*. Each experiment was performed with three biological replicates.

### GUS activity analysis

The promoters of *PtoAG1* were inserted into the *pCAMBIA2300*-GUS vector to generate *PtoAG1*-GUS. *Agrobacterium*-mediated transient transformation of 84 K poplar (*P. alba* × *P. glandulosa*) was carried out as previously described [87]. GUS staining was performed as previously reported [88]. Leaves and stems were placed in precooled 90% (v/v) acetone for 20 min and then washed with washing buffer (20 mM NaHPO_4_, pH7.2, 1 mM K_3_Fe(CN)_6_, 1 mM K_4_Fe(CN)_6_, and Triton X-100) 3 times for 2 min each time. The samples were incubated in staining solution (1 mM X-Gluc in washing buffer) at 37 ℃ overnight. Samples were cleared in 70% (v/v) ethanol.

### Electrophoretic mobility shift assay

The full-length CDSs of *PtoAG1* and *PtoMYB84* were amplified by PCR, and cloned into the expression vectors *pGEX-4 T-1* and *pET-32a*-His, respectively. PtoAG1-GST and PtoMYB84-His were purified using GST Sepharose 4FF Resin (Sangon) and Ni NTA Beads 6FF Resin (LABLEAD). EMSA was performed using an EMSA Kit (Beyotime). DNA–protein complexes were fractionated on a non-denaturing 6% polyacrylamide gel, transferred to a positive nylon membrane, and UV crosslinked. Complexes were detected using streptavidin-HRP conjugate with an Enhanced Chemiluminescence (ECL) Kit (Beyotime).

### Protein–protein interaction validation

For the Y2H assay, the coding sequences of *PtoAREB3* were inserted into the prey vector *pGADT7*, while *PtoSnRK2.2* was introduced into the bait vector *pGBKT7*, which were then co-transformed into Y2H yeast competent cells and grown on an SD-Leu-Trp medium for 2–3 days. These were then selected on a medium (SD-Trp-Leu-His-Ade-X-α-gal) for 3–5 days to examine the interactions between PtoAREB3 and PtoSnRK2.2 protein. Pairs of pGBKT7-p53 and pGADT7-T pairs served as positive controls for protein–protein interaction.

For the BiFC assay, the full-length CDSs of *PtoSnRK2.2*, *PtoAREB3*, and *PtoRR12* were ligated into the *pSPYNE(R)173* vector or *pSPYCE(MR)* vector using a ClonExpress Ⅱ One Step Cloning Kit (C112-01, Vazyme, China). *A. tumefaciens* strain GV3101 harboring individual constructs was co-transformed into leaves of *N. benthamiana* with equal volumes. After 48 h, yellow fluorescent protein (YFP) signals were observed using a laser confocal microscope (Leica SP8, Nussloch GmbH, Germany).

For the Co-IP assay, the coding sequences of *PtoSnRK2.2* were cloned into the *Super1300*-flag vector, while the full-length of *PtoRR12* was introduced into the *pGWB414*-GUS-MYC vector. The resulting constructs were individually transformed into *Agrobacterium* strain GV3101 and then co-injected into *N. benthamiana* leaves. After 3-d, the total proteins of tobacco leaves were extracted with RIPA buffer (Beyotime, P0013K) added 1 × protease inhibitor cocktail and 1 × Phosphatase Inhibitor Cocktail. 20 μL anti-flag magnetic agarose beads (Sellck) were incubated with protein samples for 12 h at 4 ℃. The beads were cleaned three times with a wash buffer ((NaCl 136.89 mM; KCl 2.67 mM; Na_2_HPO_4_ 8.1 mM; KH_2_PO_4_ 1.76 mM; 0.5% Tween20), and the immunoprecipitated proteins were separated by SDS-PAGE and detected with anti-flag and anti-MYC (1:1,000 dilution, Sigma) antibodies.

For luciferase complementation imaging (LCI) assay, the full coding regions of *PtoSnRK2.2* were inserted into the binary vector *pCambia1300-cLuc*, while the full coding regions of *PtoAREB3* were inserted into *pCambia1300-nLuc*. Agrobacterium cells harboring individual recombinant vectors were centrifuged and resuspended in infiltration medium to a final optical density at OD_600_ of 0.4 for infiltration. The resuspended solutions were mixed at a ratio of 1:1 or 1:1:1 and injected into *N. benthamiana* leaves. The plants were incubated in the dark for 12 h, followed by a 24-h incubation in a growth chamber under normal conditions. Leaves were sprayed with 100 mM luciferin and incubated in the dark for 10 min. A cooling CCD imaging apparatus (Lumazone_1300B) was used for image acquisition. Each experiment was performed in triplicate.

### Protein kinase assays

For in vitro protein expression, the recombinant proteins were purified using GST Sepharose 4FF Resin (Sangon) and Ni NTA Beads 6FF Resin (LABLEAD). PtoAREB3-GST, PtoRR12-GST and PtoMYB84-His purified fusion proteins were incubated with or without PtoSnRK2.2-GST fusion proteins in 30 μL of kinase buffer (50 mM Tris (pH 7.5), 1 mM CaCl_2_, 10 mM MgCl_2_, 1 mM dithiothreitol and 20 μM ATP) at 30 °C for 2 h. Subsequently, the reaction buffer was stopped and then separated in PAGE and for blotting with an Anti-Phosphoserine/threonine Phosphosolutions antibody (1:1,000 dilution, ECM) to detect phosphorylated bands.

Phosphorylation sites were determined by performing liquidhromatography–mass spectrometry (LC–MS/MS). To this end, the purified PtoSnRK2.2-GST, PtoAREB3-GST, and PtoAG1-GST proteins were incubated for 30 min at 30 °C in the above-mentioned phosphorylation reaction buffer before separation in SDS-PAGE. Then, the gel band containing phosphopeptides was analyzed with Thermo Scientific, Q-Exactive HF-X (BiotechPack, Beijing, China).

For in vivo protein expression, total protein was extracted from poplar leaves as previously described for *N. benthamiana* leaves. Protein concentration was determined using BCA (Beyotime) and adjusted to the same level for each sample. The specific monoclonal antibodies of PtoAREB3 were raised in rabbits (YOUKE, 1:1,000 dilution) against isoform-specific peptide SESAATSSLQRQASF. The antibody specificity was checked using PtoAREB3-GST purified fusion proteins generated from heterologous expression of PtoAREB3 in the BL21 (DE3) bacterial system. Total protein bands of PtoAREB3 were detected by western blotting using 10% (w/v) SDS-PAGE (sodium dodecyl sulfate–polyacrylamide gel electrophoresis) gel, while the phosphorylated protein bands of PtoAREB3 were detected using 10% (w/v) SDS-PAGE gel containing 40 μM phos-tag reagent and 120 μM MnCl_2_. The GAPDH band was monitored with a monoclonal antibody (ABclonal, 1:5,000 dilution). The protein bands were quantified using ImageJ software.

### Histological analyses

For the cross-sectioning and histological staining, stem cross sections of 40-μm thickness were prepared from 2-month-old WT and transgenic plants using a vibrating blade microtome (VT1000s, Leica, Wetzlar, Germany), and then stained with 0.1% (w/v) toluidine blue O (TBO) for 30 s, and were observed and captured using an Olympus DP74 digital camera (Olympus Corp., Tokyo, Japan). Image data were measured and analyzed using ImageJ software (https://imagej.nih.gov/ij/) to quantify morphological parameters of stem cells. Vessel density was calculated by dividing the total number of vessels by the entire area of the xylem region within the complete section.

For the scanning electron micrograph (SEM) observation, stem cross-Sects. (50 μm thick) were prepared from 2-month-old poplar stems, fixed in formalin-acetic acid–ethanol (FAA) solution, dehydrated through a graded ethanol series, and subjected to critical point drying for approximately 2 h. Samples were coated with gold at 10 mA for 120 s and observed under a Thermo Fisher Quanta FEG scanning electron microscope. Measurements were performed using ImageJ software on samples derived from at least three independent plants.

### Comparative genomics analyses

Homologs of the candidate genes in different *Populus* species were identified using BLAST [89] v2.2.25, and protein sequence alignments were performed using CLUSTALW2 [90] v2.1. Neighbor-Joining phylogenetic trees were constructed with MEGA-X [91]. The CODEML program in the PAML software package was utilized to calculate synonymous (Ks) and non-synonymous (Ka) substitution rates, and to estimate the divergence times of homologous gene pairs [92]. Divergence times between gene pairs were expressed in millions of years ago (Mya) using the formula T = Ks/2λ, assuming a clock-like rate (λ) of 9.0 × 10^–9^ for *Populus* [93]. Homologs of *PtoAG1* and *PtoMYB84* were identified using GeneTribe v1.2.1 [122] across six *Populus* species, as well as in woody eudicots (*Vitis vinifera*, *Malus domestica*), herbaceous eudicots (*Arabidopsis thaliana*, *Solanum lycopersicum*), a monocot (*Oryza sativa*), and gymnosperms (*Pinus densiflora*, *Ginkgo biloba*). The best homologous matches in each reference genome were selected based on default thresholds. Collinearity results were visualized using JCVI v1.5.6 [123]. Information of species genomes is provided in Supplementary Table 17.

### Statistical analyses

Quantitative data for gene expression, phenotype analyses, plant hormone contents, fluorescence intensities, luciferase activity were determined for statistical significance using Student’s *t*-test or one-way ANOVA as described in the corresponding figure legends. The piecewise structural equation modeling (SEM) was performed by using “piecewiseSEM” package. The variance inflation factor (VIF) was employed to diagnose multicollinearity among variables before SEM, with VIF value greater than 10 as the criterion for factor elimination.

## Supplementary Information

Additional file 1: Figure S1. Trait factor screening and canonical axes projection in RDA analysis. Figure S2. Parental lines and phenotypic analysis of the QTL mapping hybrid population. Figure S3. Marker density and collinearity analysis of the genetic map. Figure S4. Phenotypes of four candidate gene knockout lines within the QTL interval. Figure S5. PtoAG1 and its natural variation AG1In/Del influences the distribution and local adaptation of Populus. Figure S6. Sequence alignment and domain analysis of AG1In and AG1Del genotypes. Figure S7. Phylogenetic analysis of homologous genes of the HD-ZIP family in Populus trichocarpa, Arabidopsis thaliana and Oryza sativa with Chr7.86. Figure S8. The characterizations of stem in PtoAG1 overexpression transgenic Populus alba × Populus glandulosa. Figure S9. Schematic diagram of editing targets for knockout lines Line1 and Line34. Figure S10. Characterizations of AG1ID genotype overexpression lines in Populus. Figure S11. Morphology and physiology characteristics under drought stress of 55 cm tall transgenic plants and WT. Figure S12. Transcriptomic analysis of PtoAG1 and downstream binding assays. Figure S13. Morphology characteristics of PtoRR12-OE and PtoAREB3-OE lines under drought stress. Figure S14. The interaction assays between PtoAREB3, PtoRR12 and PtoSnRK2.2 and ABA treatment assays for WT plants. Figure S15. qPCR analysis and population transcriptome analysis of PtoSnRK2s and PtoAREBs. Figure S16. Morphology and physiology characteristics under drought stress of the ptomyb84 mutants and WT. Figure S17. Homology inference analysis among five Populus species. Figure S18. The full uncropped Gels and Blots image.

Additional file 2: Table S1. Environmental variables used in this study derived from WorldClim. Table S2. Detailed information of environmental associated variants identified in this study. Table S3. Genome-wide association signals of dynamic growth traits Table S4. Detailed information of phenotypic correlation of hybrid populations in this study. Table S5. Summary of the first 3 PCs for 11 traits in the dataset. Table S6. Detailed information of genetic map in this study. Table S7. Detailed information of significant QTLs in this study. Table S8. Genotyping for fine mapping in F1 mapping populations. Table S9. KEGG enrichment analysis of the DEGs. Table S10. DEGs and targeted genes involved in the regulation of hormone signal transduction. Table S11. Homologs of RR12 and AREB3 in the DEG data of PtoAG1 mutant. Table S12. Accession numbers of the SnRK2s genes used for BLASTP. Table S13. Motif scanning analysis of the promoter region of PtoAG1. Table S14. Information on PtoSnRK2.2-mediated phosphorylated peptides of PtoAG1 identified by LC-MS/MS analysis. Table S15. homology inference analysis in Populus and the plant taxonomic system. Table S16. Gene-specific primers used in this study. Table S17. Information of species genomes for the gene homology inference.

## Data Availability

Availability of Data and Materials The raw sequencing data of genome resequencing of 302 P. tomentosa accessions and the pseudo-testcross mapping population have been deposited in the Genome Sequence Archive in the BIG Data Center (http://bigd.big.ac.cn/gsa/) under accession numbers CRA000903 and CRA028556 [94, 95]. All transcriptome expression data reported here have been submitted to the GSA under accession nos.: CRA004084, CRA005557, CRA028513 [96–98]. The DNA affinity purification sequencing data of PtoAG1 are also available under accession no.: CRA028534 [99]. The reference genome of P. tomentosa is download from CNGB Sequence Archive (CNSA) of China National GeneBank DataBase (CNGBdb) with CNSA project ID CNP0004290 [100], and is publicly accessible at https://db.cngb.org/. Main publicly available resequencing data came from the NCBI Sequence Read Archive (SRA) (P. adenopoda (26 accessions, SRA ID: PRJNA612655 [101]), P. tremula (94 accessions, SRA ID: PRJNA297202 [102])), respectively. Main publicly available resequencing data came from the BIG Data Center (P. adenopoda (36 accessions, GSA ID: CRA001510, CRA005197 [103, 104]), P. euphratica (324 accessions, GSA ID: CRA002337 [105]), P. koreana (230 accessions, GSA ID: CRA006418 [106])), respectively. The microscopy images generated and analyzed during the current study are available in the Figshare repository (https://doi.org/10.6084/m9.figshare.32970767 [124]). The mass spectrometry proteomics data have been deposited to the ProteomeXchange Consortium via the iProX partner repository with the dataset identifier PXD081659 [125]. The 849 accessions resequencing data of P. trichocarpa came from the Joint Genome Institute under Gold study ID Gs0134612 and Gs0019845 [126, 127]. The full-length, uncropped images of all gels and blots supporting the findings of this study are provided as a supplementary file (Additional file 1: Fig. S18). The custom codes and analytical scripts used in this study are available in the GitHub repository (https://github.com/ChenfeiLv/Populus-tomentosa-population-analysis [128]) under the MIT license and on Figshare [129].
