# Supplementary material for: A molecular rheostat of ABA signaling coordinates the trade-off between stem growth and drought adaptation
Source: Genome Biol. 2026 Aug 4;27:337. doi: 10.1186/s13059-026-04228-2 (PMC13721217; doi:10.1186/s13059-026-04228-2)
Supplement: Supplementary file 1 [file 13059_2026_4228_MOESM1_ESM.docx]

**Fig. S1** Trait factor screening and canonical axes projection in RDA analysis.

**Fig. S2** Parental lines and phenotypic analysis of the QTL mapping hybrid population.

**Fig. S3** Marker density and collinearity analysis of the genetic map.

**Fig. S4** Phenotypes of four candidate gene knockout lines within the QTL interval.

**Fig. S5** *PtoAG1* and its natural variation *AG1^In/Del^* influences the distribution and local adaptation of *Populus*.

**Fig. S6** Sequence alignment and domain analysis of *AG1^In^* and *AG1^Del^* genotypes.

**Fig. S7** Phylogenetic analysis of homologous genes of the HD-ZIP family in *Populus trichocarpa*, *Arabidopsis thaliana* and *Oryza sativa* with *Chr7.86* and transactivation assay of *PtoAG1* in yeast cells.

**Fig. S8** The characterizations of stem in *PtoAG1* overexpression transgenic *Populus alba* × *Populus glandulosa*.

**Fig. S9** Schematic diagram of editing targets for knockout lines Line1 and Line34.

**Fig. S10** Characterizations of *AG1^ID^* genotype overexpression lines in *Populus*.

**Fig. S11** Morphology and physiology characteristics under drought stress of 55 cm tall transgenic plants and WT.

**Fig. S12** Transcriptomic analysis of *PtoAG1* and downstream binding assays.

**Fig. S13** Morphology and expression characteristics of *PtoRR12-OE* and *PtoAREB3-OE* lines.

**Fig. S14** The interaction assays between PtoAREB3, PtoRR12 and PtoSnRK2.2 and ABA treatment assays for WT plants.

**Fig. S15** qPCR analysis and population transcriptome analysis of *PtoSnRK2s* and *PtoAREBs.*

**Fig. S16** Morphology and physiology characteristics under drought stress of the *ptomyb84* mutants and WT.

**Fig. S17** Homology inference analysis among five *Populus* species.

**Fig. S18** The full uncropped Gels and Blots image.

**Fig. S1** Trait factor screening and canonical axes projection in RDA analysis. A, Standardized total effect size of precipitation, temperature, elevation and drought, calculated by summing the direct and indirect effects derived from the best Piecewise SEM. B, Mantel test and correlation analysis of 19 environmental variables and geographic information for RDA analysis. Five red variables indicate non-redundant variables. C, Environmental variables used in the gradient forest modeling. Variables were ordered by ranked importance. Gray column, uncorrelated environment variables (Pearson's |r| < .8). D, The first two canonical axes (RDA1 and RDA2) of the RDA of variation among 302 accessions. Each canonical axis represents a linear combination of environmental variables.

**Fig. S2** Parental lines and phenotypic analysis of the QTL mapping hybrid population. A, Phenotypic characteristics of parental lines for the hybrid population under field and greenhouse conditions. Male parent, LM50; Female parent, 1236. Bar, 15 cm. B, Correlation analysis of 11 growth traits in the hybrid population. C, 3D visualization of the PCA analysis of 11 growth traits, representing the first three PC components.

**Fig. S3** Marker density and collinearity analysis of the genetic map. A, Density of 9,675 bin markers derived from 87,562 SNPs across 19 chromosomes after binning. B, Collinearity analysis was conducted between the 19 linkage groups and the 19 chromosomes of the *Populus tomentosa* reference genome [Tianshui]. ρ denotes the collinearity rate.

**Fig. S4** Phenotypes of four candidate gene knockout lines within the QTL interval. A, Phenotypic comparison between the four candidate gene knockout lines and wild-type (WT) plants. Bar, 15 cm. B, Height of 2-mo-old plants. Error bar represent SD values of 3 biological replicates. Two-tailed paired *t*-tests, ****P* < 0.001.

**Fig. S5** *PtoAG1* and its natural variation *AG1^In/Del^* influences the distribution and local adaptation of *Populus*. A, Association testing of phenotypes (four growth traits and principal component 1 (PC1)) and 91 variants in qAG1. The x-axis represents the position in the genome. B, Geographic distribution of 302 *P. tomentosa* accessions (circles) where colors represent the natural variation *AG1^In/Del^*. The red dotted ellipse indicates the Weihe Basin region. C, left bottom represents the frequency distribution of two genotypes *AG1^In/Del^* of *PtoAG1* in three geographic populations. The box plots represent the differences among 302 *P. tomentosa* accessions in three geographical populations (upper left) and two genotypes (lower right), respectively. D, Putative sweep regions around *PtoAG1* locus were evaluated by different summary statistics. The bar denotes the location of the *PtoAG1* gene. The statistics were calculated separately for accessions from three geographic populations. southern, S; northeastern, NE; northwestern, NW. Genetic differentiation (Fst), nucleotide diversity (π), and Tajima’s D test for the presence of a selective sweep.

**Fig. S6** Sequence alignment and domain analysis of *AG1^In^* and *AG1^Del^* genotypes. The conserved HD, ZIP motif are indicated by blue arrows and green arrows, respectively; red boxed region indicates the EAR motifs; the red arrows indicate the frameshift mutation *AG1^In/Del^*; orange boxed region indicates CPSCE motif.

**Fig. S7** Phylogenetic analysis of homologous genes of the HD-ZIP family in *Populus trichocarpa*, *Arabidopsis thaliana* and *Oryza sativa* with *Chr7.86* and transactivation assay of *PtoAG1* in yeast cells. A, HD-ZIP II subfamily proteins are indicated in blue. HD-ZIP I subfamily proteins are indicated in green. HD-ZIP Ⅳ subfamily proteins are indicated in red. HD-ZIP Ⅲ subfamily proteins are indicated in orange. B, The full length of PtoAG1 was fused in frame with the GAL4 DNA-binding domain in pGBKT7 and transformed into yeast strain Y2H. The vector pGBKT7 was used as a negative control.

**Fig. S8** The characterizations of stem in *PtoAG1* overexpression transgenic *Populus alba* × *Populus glandulosa*. A, Stem diameter of internodes 3, 8, and 15 in 2-month-old plants. Bar, 5 mm. B, Xylem layers in internodes 8 and 12 of the stem. C, Cytological observations of stem cross sections of 8^th^ and 12^th^ internodes with toludine blue. Black dots indicate xylem cell layers. Error bars represent SD of 3 independent measurements. Two-tailed paired *t*-tests, **P* < 0.05, ***P* < 0.01, ****P* < 0.001.

**Fig. S9** Schematic diagram of editing targets for knockout lines Line1 and Line34. T1 and T2 denote the positions of two editing targets. The CRISPR/Cas9 genome editing system induced deletions of 168 bp and 186 bp between the two targets in the Line1 knockout line, while a 171 bp deletion between the two targets was caused in the Line34 knockout line.

**Fig. S10** Characterizations of *AG1^ID^* genotype overexpression lines in *Populus*. A, Phenotype of 8wk WT and *AG1^ID^*-OE transgenic lines (OE-1, OE-2) after transfer to soil. Bar, 15 cm. B, Height and stem diameter of 2-mo-old plants. Error bars represent SD of 3 independent measurements. Two-tailed paired *t*-tests; ns, no significance.

**Fig. S11** Morphology and physiology characteristics under drought stress of 55 cm tall transgenic plants and WT. A, The phenotype of plants under average soil relative water content (SRWC) was 65%, 22%. Bars, 15 cm. B, stem RWC and stem water potential of plants. Error bar represent SD values of 3 biological replicates. Two-tailed paired *t*-tests, **P* < 0.05, ***P* < 0.01, ****P* < 0.001. ns, no significance.

**Fig. S12** Transcriptomic analysis of *PtoAG1* and downstream binding assays. A, Volcano plots representing the fold-change of DEGs in the comparison groups of WT versus *ptoag1* (*P*＜0.05, |log₂FC| ≥ 1). Gray dots represent genes without significant changes in expression. Two independent experiments were performed for each sample. B, KEGG analysis of DEGs. C, Genes related to cytokinin synthesis and signal transduction pathways in DEGs and their fold changes in expression. D, Genes related to abscisic acid synthesis and signal transduction pathways in DEGs and their fold changes in expression. E, Enriched DNA motif identified in *PtoAG1* binding sites. E-value = 1.6e-1266. F, The KEGG analysis of DNA affinity purification sequencing (DAP-seq) for *PtoAG1*. G, EMSA of *AG1^Del^* binding to DNA probes of the *PtoAREB3* and *PtoRR12* promoters *in vitro*. The probe was biotin-labeled fragments of the *PtoAREB3* and *PtoRR12* containing the motif, whereas the cold probe was unlabeled probe (5-, 50-fold molar excess). Protein concentration was 2 µg/µL. H and I, No regulatory interaction exists between PtoAG1 and PP2Cs in the Y1H assay and the Y2H assay.

**Fig. S13** Morphology and expression characteristics of *PtoRR12-OE* and *PtoAREB3-OE* lines. A, The phenotype of *PtoRR12-OE* plants under average soil relative water content (SRWC) 68%, 19%. Bars, 15 cm. B, The phenotype of *PtoAREB3-OE* plants under average soil relative water content (SRWC) 61%, 24%. Bars, 15 cm. C, Expression profiles and correlation analysis between *PtoAG1* and *RR* homologs based on 6-year cambial transcriptome data.

**Fig. S14** The interaction assays between PtoAREB3, PtoRR12 and PtoSnRK2.2 and ABA treatment assays for WT plants. A, Y1H assays indicated that *PtoAREB3* cannot bind directly to the promoters of *PtoRR12* and *PtoRR12* cannot bind directly to the promoters of *PtoAREB3.* B, PtoRR12 and PtoAREB3 cannot interact in Y2H assays. C, Detection of phosphorylation status of PtoAREB3^S266A^ by PtoSnRK2.2. PtoSnRK2.2-GST was incubated with PtoAREB3^S266A^-GST in a kinase buffer at 30°C for 30 min. D, PtoSnRK2.2 interacts with PtoRR12 in yeast cells. E, Bimolecular fluorescence complementation assay (BiFC) showing interactions of PtoSnRK2.2 with PtoRR12 in *Nicotiana benthamiana* leaf cells. YFP signals were visualized using confocal microscopy. Bars, 25 μm. F, PtoRR12 inhibits the kinase activity of PtoSnRK2.2 *in vitro*. The phosphorylated bands were quantified using ImageJ. G, Immunoblot analysis showing an upshift of PtoAREB3 in phos-tag gels. Following 100 μM ABA treatment for 2 hours, phosphorylated PtoAREB3 protein levels were reduced in *PtoRR12-OE* lines. Total proteins were extracted from plants treated with 100 μM ABA or mock control for 2 h. PtoAREB3 and GAPDH separated on standard SDS-PAGE gels (without Phos-tag) were detected as loading controls. H, Relative expression levels of *PtoAG1* and *PtoAREB3* in WT. 1-mo-old plants were treated with 50 μM ABA for 1, 2, 4, 6 and 12, followed by total RNA extraction. I, Relative expression levels of PtoRR12 in WT, *ptoag1* and *PtoAG1-OE*. 1-mo-old plants were treated with 100 μM ABA for 0.5, 1, 2, 4, and 8 h, followed by total RNA extraction. 3 independent experiments were performed for each sample. Data represent means SD of 3 independent experiments. Error bars are overlaid by symbols when not visible.

**Fig. S15** qPCR analysis and population transcriptome analysis of *PtoSnRK2s* and *PtoAREBs.* A, Dynamic transient expression profiles of *PtoSnRK2s* under ABA, dehydration, and fluridone+dehydration conditions (0-24 h). B and C, Correlation analysis of population-level transcriptomics (transcriptome data of a germplasm population consisting of 50 *Populus tomentosa* individuals before and after drought stress) showed coordinated *PtoSnRK2.2* and *PtoAREB3* induction during drought and post-recovery dynamics.

**Fig. S16** Morphology and physiology characteristics under drought stress of the *ptomyb84* mutants and WT. A, The phenotype of plants under average soil relative water content (SRWC) was 65%, 21%. Bars, 15 cm. B, stem RWC and stem water potential of plants. Error bar represent SD values of 3 biological replicates. Two-tailed paired *t*-tests, **P* < 0.05, ***P* < 0.01. ns, no significance.

**Fig. S17** Homology inference analysis among five *Populus* species. Blue connections represent *MYB84* orthologs and orange connections represent *AG1* orthologs. The physical distance on the left represents the total distance of the selected fragments.

**Fig. S18** The full uncropped Gels and Blots image.
